# Temporal Association Cortex Gates Sound‐Evoked Arousal from NREM Sleep

**DOI:** 10.1002/advs.202414271

**Published:** 2025-01-31

**Authors:** Haipeng Yu, Jincheng Wang, Ruiqi Pang, Penghui Chen, Tiantian Luo, Xuan Zhang, Yatao Liao, Chao Hu, Miaoqing Gu, Bingmin Luo, Zhiyue Shi, Mengyao Li, Yueting Zhang, Qiaoqian Wei, Wei Yuan, Hui Xie, Zhiyi Chen, Hongbang Liu, Shuancheng Ren, Xiaowei Chen, Yi Zhou

**Affiliations:** ^1^ Advanced Institute for Brain and Intelligence School of Physical Science and Technology Guangxi University Nanning 530004 China; ^2^ Department of Neurobiology College of Basic Medicine Army Medical University Chongqing 400038 China; ^3^ Guangxi Key Laboratory of Special Biomedicine School of Medicine Guangxi University Nanning 530004 China; ^4^ Department of Neurosciences Case Western Reserve University School of Medicine Cleveland OH 44106 USA; ^5^ Department of Otolaryngology Chongqing General Hospital Chongqing University Chongqing 400038 China; ^6^ School of Architecture and Urban Planning Chongqing University Chongqing 400044 China; ^7^ Experimental Research Center for Medical and Psychological Science School of Psychology Army Medical University Chongqing 400038 China; ^8^ Department of Physiology College of Basic Medicine Army Medical University Chongqing 400038 China; ^9^ Brain Research Center and State Key Laboratory of Trauma and Chemical Poisoning College of Basic Medicine Army Medical University Chongqing 400038 China

**Keywords:** arousal, basolateral amygdala, lateral amygdala, NREM sleep, sound, temporal association cortex

## Abstract

Sound‐evoked wakefulness from sleep is crucial in daily life, yet its neural mechanisms remain poorly understood. It is found that CaMKIIα+ neurons in the temporal association cortex (TeA) of mice are not essential for natural awakening from sleep. However, optogenetic activation of these neurons reliably induces wakefulness from non‐rapid eye movement (NREM) sleep but not from rapid eye movement (REM) sleep. In vivo electrophysiological and calcium recordings further demonstrated that TeA neurons are monotonically tuned to sound intensity but not frequency. More importantly, it is found that the activity of CaMKIIα+ neurons in TeA can gate sound‐evoked arousal from NREM sleep, which is further confirmed by optogenetic manipulations. Further investigation reveals that the baseline excitability of TeA CaMKIIα+ neurons and the delta oscillations in the electroencephalogram are particularly important in regulating the evoked activity of TeA neurons. Anatomical and functional screening of downstream targets of TeA reveals that excitatory projections from TeA glutamatergic neurons to glutamatergic neurons in the basolateral/lateral amygdala are critical for modulating sound‐evoked arousal from NREM sleep. These findings uncover a top‐down regulatory circuit that selectively governs sound‐evoked arousal from NREM sleep, with the TeA functioning as a key connecting cortex to subcortical regions.

## Graphical abstract

It is often wondered why some sounds wake people while others don't. Researchers have discovered that the temporal association cortex plays a key role in this during NREM sleep. Neurons in this region work with the amygdala to decide which sounds are important enough to interrupt sleep, providing a balance between rest and responsiveness.

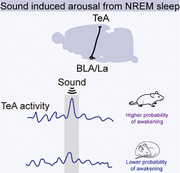

## 1 Introduction

Sound‐evoked arousal, the most common form of externally inducing sleep‐wake transitions, occurs when auditory stimuli, such as noises, trigger the brain to shift from a state of sleep to wakefulness.^[^ ^1^, ^2^, ^3^ ^]^ This arousal mechanism serves as a protective response, enabling organisms to react to potential threats or important environmental changes,^[^ ^4^, ^5^, ^6^, ^7^ ^]^ such as thunder, an alarm clock, or a crying baby. These sounds can disrupt sleep by activating the auditory related pathways in the brain, prompting an individual to become alert and responsive to their environment.^[^ ^7^, ^8^ ^]^ Understanding the neural mechanisms underlying sound‐evoked arousal from sleep is crucial for developing effective strategies to improve sleep quality and mitigate the negative impacts of noise on sleep.^[^ ^1^, ^9^, ^10^ ^]^

Mechanistically, sound‐evoked arousal is mediated by intricate neural pathways involving both the auditory systems responsible for auditory information processing^[^ ^11^, ^12^ ^]^ and the brain centers responsible for regulating sleep and wakefulness^[^ ^8^, ^13^ ^]^ When an auditory stimulus is detected, it is processed by the cochlea in the inner ear, which converts sound waves into neural signals.^[^ ^14^, ^15^ ^]^ These signals are then transmitted to the auditory cortex via the auditory nerve and brainstem.^[^ ^12^, ^16^ ^]^ If the sound is of sufficient intensity^[^ ^17^, ^18^ ^]^ or has particular significance,^[^ ^7^, ^8^ ^]^ the arousal centers are engaged, leading to cortical arousal and transition from sleep to wakefulness. Recent studies have shown that certain brain regions, such as the thalamus^[^ ^7^, ^19^ ^]^ and brainstem,^[^ ^8^, ^20^ ^]^ play critical roles in modulating the brain arousal to different sounds. However, the precise role of the cortex in mediating sound‐evoked arousal from sleep has been less understood.

The temporal association cortex (TeA) is a cortical area adjacent to the ventral part of the auditory cortex in rodents (Allen Brain Atlas), often defined as a multisensory hub that integrates auditory, visual, and somatic inputs.^[^ ^21^, ^22^, ^23^ ^]^ Recent studies have shown that it receives extensive inputs from both the auditory thalamus and the primary auditory cortex, suggesting that TeA plays an important role in auditory processing.^[^ ^7^, ^24^ ^]^ For example, TeA has been found to be critical for vocal communication between mother and pup mice, which is essential for maternal care.^[^ ^25^ ^]^ In this study, we investigated the neural mechanisms underlying sound‐evoked arousal in mice, focusing on the role of the TeA. We found that TeA CaMKIIα+ neurons can selectively gate sound‐evoked arousal from non‐rapid eye movement (NREM) sleep through excitatory projections to downstream neurons in the basolateral/lateral amygdala (BLA/La), revealing a top‐down regulation of sound‐evoked arousal.

## 2 Results

### 2.1 TeA Activation Selectively Facilitates Arousal From NREM Sleep in an Unconventional Way

**Figure 1:**
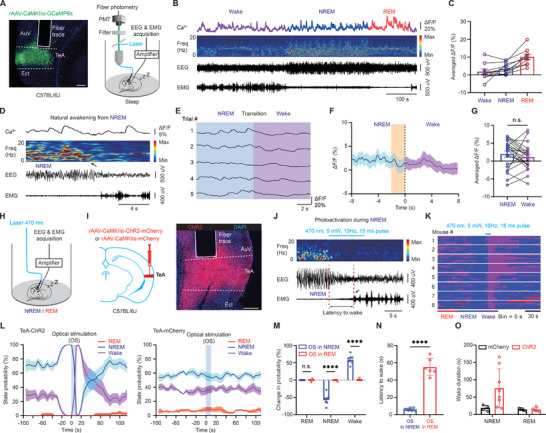
Role of TeA CaMKIIα+ neurons in the transition from NREM sleep to wake. A) Left: GCaMP6s expression and fiber trace in the TeA. Right: Schematic of monitoring of the Ca^2+^ signal of TeA CaMKIIα+ neurons and sleep/wake state by recording EEG/EMG activity. TeA: temporal association cortex; AuV: ventral area of auditory cortex; Ect: ectorhinal cortex. Scale bar, 200 µm. B) Ca^2+^ activity during natural sleep/wake changes. Colored map represents the power (mV^2^) of the raw power spectral density. Freq, frequency. C) Averaged Ca^2+^ activity in different states, 10 traces from 6 mice. D) Example of NREM‐wake transition (black arrow). E) Five representative Ca^2+^ activities during the NREM‐wake transition. Blue and purple shading represent NERM and wake states, respectively. F) Averaged Ca^2+^ activity during the NREM‐wake transition, 22 transitions from 6 mice. The dotted line indicates the NREM‐wake transition. The translucent yellow indicates neuronal activity 2 s before the transitions. G) Comparison of averaged ΔF/F values within 8s before and after the transition, n = 6 mice. H) Schematic of optogenetic manipulation with simultaneous EEG‐EMG monitoring during sleep. I) Left: Schematic of virus injection into the TeA. Right: ChR2‐mCherry expression and location of the optical fiber in the TeA. Scale bar, 200 µm. J) Representative sleep‐wake changes after 10 Hz optogenetic stimulation during NREM sleep. Red dotted lines indicate the latency between the light onset and wakefulness as indexed by the EMG change (black arrow). K) Photoactivation results from all trials in ChR2‐expressing mice. L) Left: Probability of each state around optogenetic stimulation during NREM sleep in the ChR2 group, n = 8 mice. Right: control group (mCherry), n = 4 mice. Rectangular shading represents the optogenetic stimulation (OS). M) Probability change of each state 10‐s before and after stimulation onset during NREM or REM sleep. N) Latency from stimulation onset to wake. O) Duration from first wake after OS to next sleep. ****, *P* < 0.0001; n.s., not significant. Error bars represent SEM. See Table S1 (Supporting Information) for details on statistical data analysis.

We used EEG(electroencephalogram) and EMG (electromyogram) recordings to identify sleep states and measure calcium signals from CaMKIIα+ neurons in the TeA, assessing their spontaneous activity in adult mice (**Figure** **1**A). A high level of activity was observed during all sleep and wake states (Figure 1B), with a particularly pronounced occurrence during rapid eye movement (REM) sleep (Figure 1C). During natural awakening from NREM sleep (Figure 1D), no significant calcium transients were observed in TeA (Figure 1E). Both the averaged trace of the calcium signal (Figure 1F) and the statistical comparisons between the amplitude of the calcium signal before and after awakening showed no significant change (Figure 1G), suggesting that these neurons may not be actively involved in natural awakening from NREM sleep. Unexpectedly, optogenetic activation (Figure 1H,I; Figure S1, Supporting Information) of these neurons led to rapid wakefulness from NREM sleep (Figure 1J), as evidenced by reliable NREM‐wake transitions across different trials, changes in state probability, decreased latency to wake (indexed by EMG change), and increased wake duration (Figure 1K–N). No such change was observed following optogenetic activation in the control group (Figure 1L) or in REM sleep (Figure 1M–O). These results suggest that the TeA may play a distinct role in sleep‐wake state transitions compared to subcortical areas such as the paraventricular thalamus (PVT), ventral tegmental area (VTA), lateral hypothalamus (LH), and locus coeruleus (LC), which are key regulators of the sleep‐wake state transition and show increased activity during natural awakening.^[^ ^26^, ^27^, ^28^, ^29^, ^30^, ^31^ ^]^

Moreover, although an apparent decrease in calcium activity is observed during natural awakening from REM sleep (Figure S2A–C, Supporting Information), spontaneous increases or decreases in calcium activity during REM sleep with even much larger amplitude changes do not trigger arousal from REM sleep (Figure S2D,E, Supporting Information). Optogenetic activation of TeA neurons also fails to induce transitions between sleep and wake states or between different sleep phases during REM sleep (Figure S2, F‐J). This suggests that the observed reduction in calcium activity during the transition from REM sleep to wakefulness is not the cause of awakening, but rather a consequence of the sleep‐wake state transition, as shown in Figure 1B.

In addition, chemogenetic inhibition of the TeA using rAAV‐CaMKIIα‐hM4Di‐mCherry combined with CNO(Clozapine N‐oxide) administration selectively resulted in increased NREM sleep and decreased wakefulness, without affecting REM sleep (Figure S3, Supporting Information). These findings strongly indicate that TeA CaMKIIα+ neurons function as a critical node in the transition to arousal from NREM sleep. Although these neurons may not play a role in natural awakening, they possess the capacity to influence arousal when appropriately stimulated.

### 2.2 TeA CaMKIIα+ Neurons Gate Sound‐Evoked Arousal From NREM Sleep

Given the anatomical proximity of the TeA to the auditory cortex and its involvement in auditory processing,^[^ ^24^ ^]^ we hypothesized that TeA CaMKIIα+ neurons might play a role in sound‐evoked arousal from NREM sleep. We first investigated the response characteristics of TeA neurons to different types of sound. In vivo patch‐clamp recordings (Figure S4A, Supporting Information) were performed to record spike activities from single neurons in head‐fixed awake mice. Our in vivo patch‐clamp recordings revealed that TeA pyramidal neurons exhibit a monotonic response to the sound pressure levels of both white noise (R^2^ = 0.5918; Figure S4B,C, Supporting Information) and pure tones (R^2^ = 0.6558; Figure S4D–F, Supporting Information). However, these neurons are poorly tuned to the frequency of pure tones (R^2^ = 0.0776; Figure S4G), indicating a sensitivity to sound intensity rather than frequency. Fiber photometry recordings in awake mice from TeA CaMKIIα+ neurons labeled with rAAV2/9‐CaMKIIα‐GCamp6s (Figure S4H,I, Supporting Information) also showed that calcium signals are positively correlated with the sound pressure levels (R^2^ = 0.4458; Figure S4J,K, Supporting Information). These results indicate that the activity of TeA neurons is tuned to sound pressure levels in awake mice, suggesting that the TeA may play a role in sound‐evoked arousal from NREM sleep.

**Figure 2:**
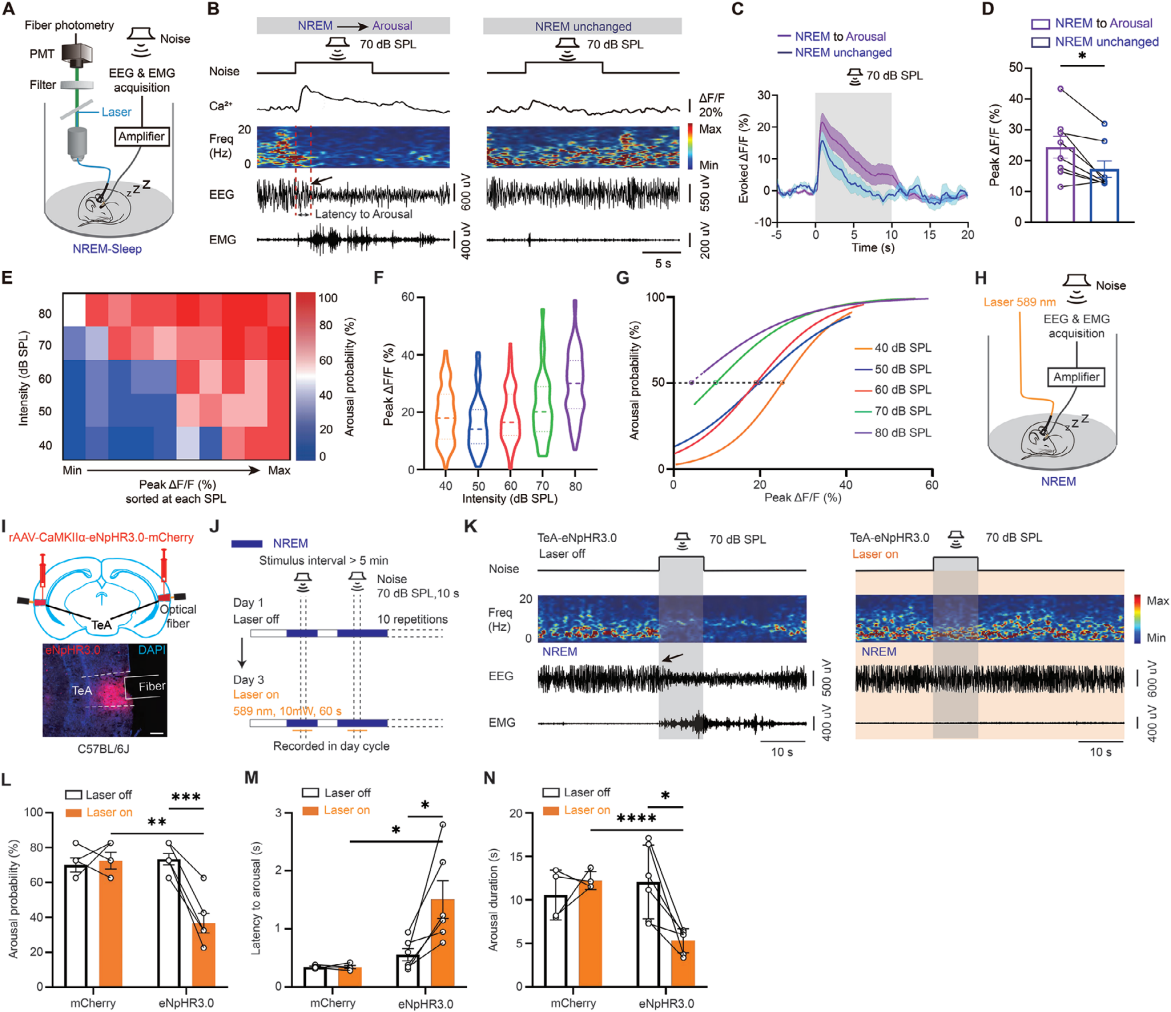
Role of TeA CaMKIIα+ neurons in sound‐evoked arousal from NREM sleep. A) Schematic of monitoring of TeA CaMKIIα+ neuron Ca^2+^ activity and sleep/wake state to sound stimuli. B) Representative case where 70 dB SPL noise can evoke arousal (left) and cannot evoke arousal (right). Red dotted lines indicate the latency between the sound onset and arousal as indexed by the EEG change (black arrow). C) Averaged sound‐evoked Ca^2+^ activity of TeA CaMKIIα+ neurons during NREM sleep, in both aroused and non‐aroused cases. D) Comparison of sound‐evoked Ca^2+^ activities in aroused and non‐aroused cases. n = 8 mice. E) Relationship between sound intensity, sound‐evoked Ca^2+^ activity, and arousal probability. F) The distribution of sound‐evoked Ca^2+^ activity at different sound intensities. G) Logistic regression curves of calcium amplitudes and arousal probability at different sound intensities. Circles indicate calcium amplitudes associated with a 50% probability of arousal (dashed line). H) Schematic of optogenetic inhibition (left). I) Schematic of virus injection into the TeA (top) and virus expression and fiber track (bottom). Scale bar, 200 µm. J) Protocol for the delivery of optical and sound stimuli. K) Representative sleep/wake changes to 70 dB SPL noise during NREM sleep without (left) or with (right) optical stimulation. L) Probability of sound‐evoked arousal. mCherry, 4 mice; eNpHR3.0, 6 mice. M) Latency from noise onset to arousal. N) Duration from the first wake after noise to next sleep. *, *P* < 0.05; **, *P* < 0.01; ***, *P* < 0.001; ****, *P* < 0.0001; n.s., not significant. Error bars represent SEM. See Table S1 (Supporting Information) for details on statistical data analysis.

We then delivered white noise at varying sound pressure levels and used EEG and EMG to identify sleep states, while recording calcium signals from CaMKIIα+ neurons in the TeA to investigate their role in sound‐evoked arousal in mice (**Figure** **2**A). Clear sound‐evoked calcium signals can be observed (Figure 2B,C; Figure S5A–D, Supporting Information). Notably, significantly higher sound‐evoked calcium activity of TeA CaMKIIα+ neurons was detected when the sound evoked arousal from NREM sleep compared to non‐arousal conditions (Figure 2C,D, Supporting Information), suggesting that TeA activity is associated with sound‐evoked arousal from NREM sleep. No such difference was observed during sound‐evoked arousal from REM sleep (Figure S5E–I, Supporting Information).

To better understand the role of TeA CaMKIIα+ neurons in sound‐evoked arousal, Figure 2E presents a detailed analysis of the relationship between sound‐evoked calcium activity, sound pressure level, and arousal probability from NREM sleep. As expected, higher sound pressure levels increase the likelihood of arousal, reflecting the general principle that louder sounds are more likely to induce arousal in a sleeping animal. However, the role of sound‐evoked calcium activity in this process is more nuanced. Since TeA activity varies with sound, we need to analyze the amplitude of calcium signals at different sound levels to understand the connection between TeA activity and the likelihood of sound‐evoked arousal during NREM sleep. An intriguing observation is that even at relatively low sound pressure levels, such as 40 or 50 dB SPL, there remains a high probability of arousal, provided that the activity level of the TeA neurons is high. Conversely, at high sound pressure levels, such as 70 or 80 dB SPL, the probability of arousal decreases when TeA neuron activity is low. These data underscore the critical role of TeA CaMKIIα+ neuron activity in selectively altering the threshold or sensitivity of the sleeping brain to external sounds during NREM sleep, similar to the role of a sentinel.

To further clarify how TeA CaMKIIα+ neuron activity contributes to sound‐evoked transitions from NREM sleep to arousal, we first examined the distribution of calcium responses at different sound pressure levels during NREM sleep. The results reveal that the amplitude of TeA response rises as intensity increases (Figure 2F), which is expected. In addition, we performed logistic regression analyses at multiple sound intensities and identified the calcium amplitudes associated with a 50% probability of arousal at the midpoint of these regression curves (Figure 2G). We found that the calcium amplitude at the midpoint decreased with increasing sound intensity, suggesting that louder sounds reduce the level of TeA activity required to induce arousal. The S‐shaped logic regression curve at low sound pressure levels and the non‐S‐shaped regression curve at high sound pressure levels also support the possibility of a dynamic threshold related to sound intensity in sound‐evoked arousal. Taken together, these results suggest that louder sounds both increase the activity level of TeA neurons and lower the activity threshold required to achieve arousal, leading to a nonlinear rapid increase in the probability of arousal, which helps to explain why more intense sounds lead to easier arousal.

Since optogenetic activation may directly induce arousal, we chose to further verify the gating effect of TeA with optogenetic inhibition (Figure 2H–N). Optogenetic inhibition of TeA CaMKIIα+ neurons with eNpHR3.0 also confirmed the gating effect of TeA (Figure 2L), as evidenced by reduced arousal probability (Figure 2L), increased latency to arousal (indexed by EEG change, Figure 2M), and reduced arousal duration (Figure 2N) following 70 dB SPL white noise stimulation.

### 2.3 Baseline Excitability and Low Frequency EEG Modulate Activity of TeA CaMKIIα+ Neurons

**Figure 3:**
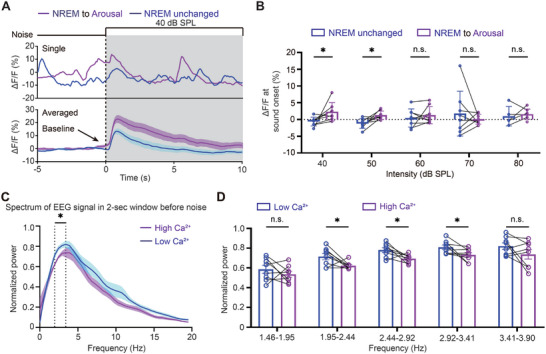
Baseline excitability and low frequency EEG modulate activity of TeA CaMKIIα+ neurons. A) Example (top) and averaged trace (bottom) of Ca^2+^ activity evoked by 40 dB SPL noise during NREM sleep, in aroused and non‐aroused cases. B) Comparison of baseline activity from 40 to 80 dB SPL noise, in both aroused and non‐aroused cases. n = 8 mice. C) Comparison of the normalized power spectrum of EEG signals within a 2‐second window preceding noise, followed by either high or low Ca^2+^ activity. D) A detailed comparison of EEG power spectrum from 1.46–3.90 Hz shown in C. *, *P* < 0.05; **, *P* < 0.01; ***, *P* < 0.001; n.s., not significant. Error bars represent SEM. See Table S1 (Supporting Information) for details on statistical data analysis. Arrow indicates the arousal evoked by sound. Gray shading indicates sound stimulation.

The above results suggest that both sound pressure level and sound‐evoked TeA activity level jointly determine the likelihood of sound‐evoked arousal from NREM sleep. However, since TeA activity is also influenced by sound pressure level, it is necessary to further clarify whether TeA activity levels alone can independently influence sound‐evoked arousal. We measured the transient activity level of TeA (**Figure** **3**A) just before the sound onset to observe the influence of TeA neuron excitability on the probability of sound‐evoked arousal. Notably, a higher level of baseline activity of TeA CaMKIIα+ neurons prior to the sound was found when 40 or 50 dB SPL white noise evoked arousal compared to non‐arousal conditions (Figure 3A,B), suggesting that the TeA's excitability alone can modulate the responsiveness of the brain to external sound during NREM sleep.

However, no such difference in baseline activity was observed when loud white noise was delivered (60–80 dB, Figure 3B). This may be because the high sound pressure levels dominate the influence on arousal probability, overshadowing the contribution of TeA CaMKIIα+ neuron excitability. Therefore, factors beyond baseline excitability levels are likely to play a more significant role in regulating TeA neuronal activity during NREM sleep. Given that TeA activity is strongly correlated with different brain states, as shown in Figure 1B, and that EEG is commonly used as an indicator of brain state, we next examined the relationship between the brain state preceding the sound and the sound‐evoked activity of TeA CaMKIIα+ neurons. For each animal, the power spectrum of the EEG signals during the 2‐s window preceding the noise onset was calculated (Figure 3C) and classified into two groups: those followed by the highest calcium activities and those followed by the lowest calcium activities, both evoked by the same sound over different repetitions. Since this grouping method is independent of the sound pressure level, we compared the two groups of EEG spectrum over all animals and all sound pressure levels (Figure 3C). Significantly higher power in continuous low frequency bands (1.95–3.41 Hz, delta oscillation) is associated with relatively lower sound‐evoked calcium activity (Figure 3D). These results suggest that both baseline excitability and low‐frequency EEG modulate the response activity of TeA CaMKIIα+ neurons to external sounds, which is critical for sound‐evoked arousal from NREM sleep.

### 2.4 Screening of Downstream Targets of TeA Responsible For Sound‐Evoked Arousal

**Figure 4:**
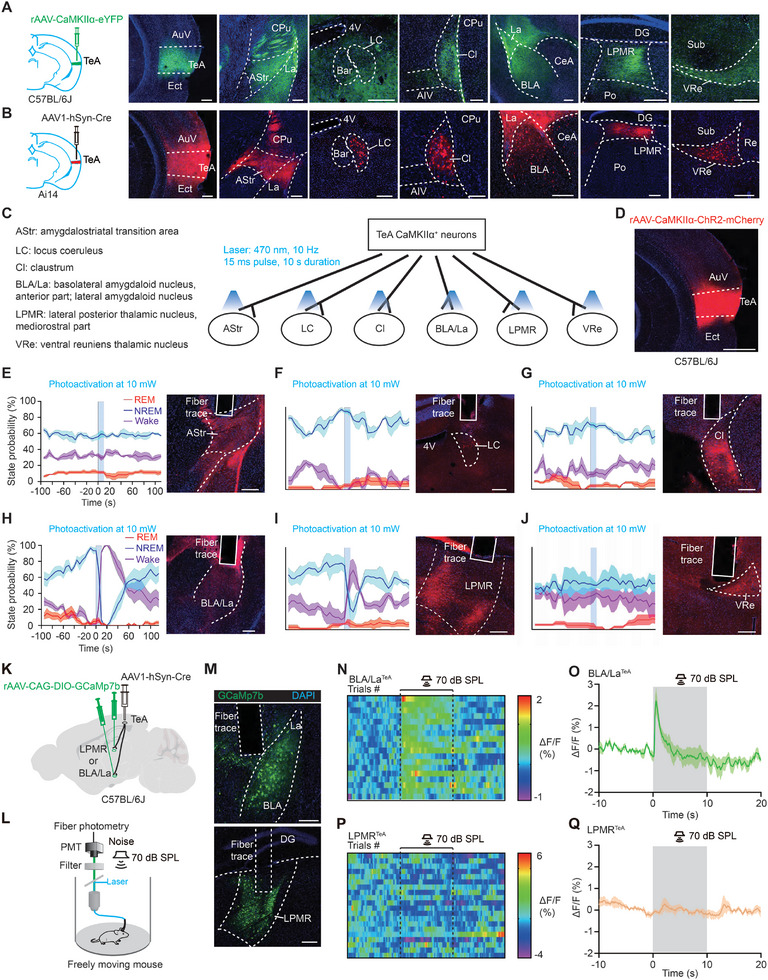
Anatomical and functional screening of downstream targets of TeA responsible for sound‐evoked arousal. A,B) Left: Schematic of non‐trans‐synaptic (A) and trans‐synaptic (B) anterograde viral tracing. Right: Representative fluorescence of injecting site in the TeA and eYFP (A)/Tdtomato (B) expression in downstream targets. Scale bars, 200 µm. C) Schematic of photoactivation on the downstream targets of TeA CaMKIIα+ neurons. D) A representative ChR2 expression in the TeA. Scale bar, 500 µm. E–J) shows the change of sleep/wake states after optogenetic stimulation in different downstream targets of TeA CaMKIIα+ neurons (left) and the expression of tdTomato (right). (E) AStr: 4 mice; (F) LC: 4 mice; (G) Cl: 4 mice; (H) BLA/La: 6 mice; (I) LPMR: 4 mice; (J) VRe: 5 mice. (K) and (L): schematic of virus injection (K) and experimental design (L) for recording calcium activity of neurons in BLA/La and LPMR receiving synaptic input from TeA. M) GCaMP7b expression and fiber trace in BLA/La (top) and LPMR (bottom), respectively. Scale bar, 200 µm. Representative heatmaps N) and averaged O) sound‐evoked Ca^2+^ activity of BLA/La neurons receiving input from TeA, n = 5 mice. Representative heatmaps P) and averaged sound‐evoked Ca^2+^ activity (Q) of LPMR neurons receiving input from TeA, n = 4 mice. The dotted lines in (N) and (P) and the gray shading in (O) and (Q) indicate the onset and offset of the 70 dB noise.

We then examined the downstream targets of TeA neurons. Both non‐trans‐synaptic (**Figure** **4**A) and trans‐synaptic (see Methods for details, Figure 4B) tracing of TeA neurons showed that TeA neurons can project to multiple brain targets. We have selected some of the most prominent regions based on the intensity and extent of fluorescence, including AStr (amygdalostriatal transition area), LC (locus coeruleus), Cl (claustrum), BLA (basolateral amygdala), La (lateral amygdala), LPMR (lateral posterior thalamic nucleus, mediorostral part) and VRe (ventral reuniens thalamic nucleus).

Next, we performed functional verification with projection specificity by optogenetically activating the axon terminals of TeA‐projecting neurons in the downstream regions (Figure 4C). These axon terminals were expressed with ChR2 by injecting AAV‐CaMKIIα‐ChR2‐mCherry into the TeA (Figure 4D). Among all the downstream targets, optogenetic activation of axon terminals revealed that only terminal activation in the BLA/La and LPMR can induce arousal from NREM sleep (Figure 4E–J and Figure S6A, Supporting Information), similar to the effect observed when TeA neurons were optogenetically activated.

Considering that the downstream targets we are looking for should be sensitive to sound, we examined c‐Fos expression in BLA/La and LPMR in freely moving mice after noise exposure (70 dB, 90 min, Figure S6B, Supporting Information) while the mice were awake. C‐Fos labeling revealed that increased c‐Fos expression can be found in the TeA (Figure S6C) and BLA/La regions (Figure S6D, Supporting Information), but not in the LPMR after noise exposure (Figure S6E, Supporting Information), suggesting that the LPMR may not play a significant role in auditory processing. Using an anterograde transynaptic labeling strategy (Figure 4K–M) similar to that in Figure 4B, we further examined the calcium activities of neurons in BLA/La and LPMR receiving projections from TeA to 70 dB noise stimulation. It is found that a strong and reliable sound‐evoked calcium response can be observed in BLA/La (Figure 4N–O) but not in LPMR regions (Figure 4P–Q), in line with the c‐Fos results, suggesting that the TeA‐LPMR pathway may not be involved in sound‐evoked arousal. These results suggest that unlike TeA neurons or BLA/La neurons receiving input from TeA, which share similar characteristics, LPMR neurons receiving input from TeA are unlikely to be involved in sound evoked arousal.

Together with the optogenetic activation and calcium recording results, these findings suggest that the TeA‐BLA/La pathway may act as a primary modulator of sound evoked arousal.

### 2.5 TeA^Glut^‐BLA/La^Glut^ Pathway Selectively Regulates Sound‐Evoked Arousal From NREM Sleep

**Figure 5:**
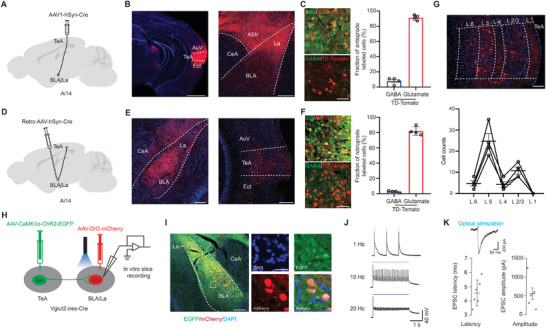
Morphology and function dissection of TeA‐BLA/La circuit. A) Schematic of TeA injection of AAV1‐Cre into Ai14 mice. B) Viral expression (Td‐Tomato) at the injection site (left) and in the BLA/La area. Scale bar, left: 1 mm; right: 200 µm. C) Left: Representative co‐localization of glutamatergic neurons and TdTomato+ neurons (top), and GABAergic neurons and TdTomato+ neurons (bottom). Scale bar, 20 µm. Right: Percentage of antegrade labeled cells. D) Schematic of BLA/La injection of retroAAV‐Cre into Ai14 mice. E) Viral expression (TdTomato) at the injection site (left) and in the TeA area. Scale bar, left, 1 mm; right, 200 µm. F) Left: Representative co‐localization of glutamatergic neurons and TdTomato+ neurons (top), and GABAergic neurons and TdTomato+ neurons (bottom). Scale bar, 20 µm. Right: Percentage of retrograde labeled cells. G) Top: A zoomed‐in view of the TeA shown in E, with laminar labels. Bottom: Counts of TeA neurons projecting to the BLA/La region in different cortical layers. A total of 178 cells were counted from 4 mice. Scale bar, 100 µm. H) Schematic of patch clamp recording to study the role of TeA CaMKIIα+ neuron terminals labeled by AAV‐CaMKII‐ChR2‐EGFP on BLA/La VGlut^2+^ neurons labeled by AAV‐DIO‐mCherry in acute brain slices. I) Representative viral labeling of TeA CaMKIIα+ neuron terminals (EGFP) and Vglut^2+^ neurons (mCherry) within the BLA/La. Scale bars, 200 µm (left) or 20 µm (right). J) Representative excitatory postsynaptic potentials (EPSPs) and action potentials evoked by blue light (470 nm, 15 mW, 15 ms, blue line) recorded from BLA/La mCherry‐expressing neurons in acute brain slices. K) Top: Representative excitatory postsynaptic currents (EPSCs) evoked by optical stimulation. Bottom: Latency and amplitude of EPSCs evoked by optical stimulation (470 nm, 15 mW, 15 ms).

To further clarify the upstream and downstream cell types in the TeA‐BLA/La pathway, we combined viral tracing with immunohistology (**Figure** **5**). The results showed that the majority of the neurons in the BLA/La receiving input from TeA are glutamatergic neurons (91.25%, Figure 5A–C), while ≈8.00% are GABAergic neurons. In addition, ≈82.00% of the neurons in TeA that project to the BLA/La region are also glutamatergic neurons (Figure 5D‐F), and about 2.75% are GABAergic neurons. In addition, the retrogradely labeled neurons in the TeA, including a portion of the ectorhinal cortex (Ect), were located primarily at a depth of ≈200 to 800 µm, corresponding to a localization from layer 2 to layer 5^[^ ^32^, ^33^ ^]^ (Figure 5G). In vitro patch clamp recordings (Figure 5H,K) in brain slices further confirmed these findings, as spikes (Figure 5J) and large EPSCs (Figure 5K) were observed in BLA/La glutamatergic neurons when terminals of TeA CaMKIIα+ neurons were optogenetically activated. These results suggest that the majority of upstream and downstream neurons in the TeA‐BLA/La pathway are excitatory glutamatergic neurons, which is consistent with the findings in Figure 1, as CaMKIIα+ neurons are predominantly excitatory glutamatergic neurons in the cortex.^[^ ^34^, ^35^ ^]^

**Figure 6:**
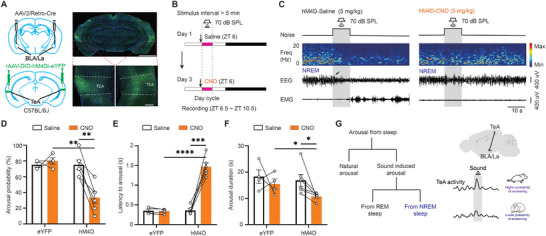
TeA‐BLA/La circuit is essential for sound‐evoked arousal from NREM sleep. A) Left: schematic of virus injection for chemogenetic inhibition. Right: expression of hM4Di‐eYFP in the TeA. Scale bar, 2 mm (top) or 200 µm (bottom). B) Protocol for chemogenetic manipulations and sound stimulation. C) Representative sleep/wake changes to 70 dB SPL noise during NREM sleep with saline injection (left) or with CNO injection (right). Arrow indicates the arousal evoked by sound. Panels (D–F) represent the probability of arousal, latency to arousal, and duration of arousal in saline and CNO trials, respectively. hM4D, n = 6 mice; eYFP, n = 4 mice. G) Diagram of the gating role of the TeA‐BLA/La circuit in sound‐evoked arousal from NREM sleep. *, *P* < 0.05; **, *P* < 0.01; ***, *P* < 0.001; ****, *P* < 0.0001; n.s., not significant. Error bars represent SEM. See Table S1 (Supporting Information) for details on statistical data analysis.

The role of the TeA‐BLA/La pathway in sound‐evoked arousal from NREM sleep was then examined. Chemogenetic inhibition of the TeA‐BLA/La pathway (**Figure** **6**A,B) lead to the suppression of sound‐evoked arousal from NREM sleep (Figure 6C). This was evidenced by a reduction in arousal probability (Figure 6D), an increase in latency to arousal (indexed by EEG change, Figure 6E), and a decrease in wakefulness duration (Figure 6F). Moreover, sound‐evoked arousal from NREM sleep is also correlated with the activity level of the TeA‐BLA/La pathway (Figure S7, Supporting Information). These findings reveal a gating mechanism through which the TeA modulates the brain's sensitivity to external auditory stimuli during sleep, thereby advancing our understanding of sensory processing in sleep‐wake transitions.

## 3 Discussion

In this study we demonstrate that TeA CaMKIIα+ neurons do not significantly change their activity during natural awakenings but are essential for modulating sound‐evoked arousal from NREM sleep through a TeA‐BLA/La glutamatergic circuit. This mechanism fine‐tunes how the brain responds to external auditory stimuli during sleep, providing new insights into sensory processing in sleep‐wake transitions. Moreover, our data suggest that REM sleep may rely on separate neural pathways for sound‐induced arousal.

### 3.1 Integration with Existing Arousal Mechanisms

Previous studies have identified key subregions in the thalamus and brainstem—such as the auditory thalamus,^[^ ^7^ ^]^ mediodorsal thalamus,^[^ ^19^ ^]^ locus coeruleus,^[^ ^20^ ^]^ and pontine central gray^[^ ^8^ ^]^—that are critical for sound‐evoked arousal. A common feature of these areas is that they are involved in both natural and sensory‐evoked arousal, consistent with their role as part of the broader reticular activating system in the brain.^[^ ^30^ ^]^ By contrast, our findings highlight the TeA's selective involvement in sound‐induced awakening from NREM sleep only. TeA was found to receive synaptic inputs from subcortical regions like the medial part of auditory thalamus,^[^ ^7^ ^]^ as well as from other cortical regions like auditory cortex.^[^ ^25^ ^]^ Thus, this mixed anatomical and functional basis provides TeA with a great deal of possibility and flexibility to integrate auditory processing capabilities from both sides.

In this study, we found that the activities of TeA CaMKIIα+ neurons were most active during REM sleep than during NREM sleep and wakefulness. However, activation of these neurons promotes transitions from NREM sleep to wakefulness, but not from REM sleep to wakefulness. This observation is consistent with previously reported activity profiles and functional roles of some populations of wakefulness‐promoting neurons. For example, GABAergic neurons in the lateral hypothalamus^[^ ^36^ ^]^ and cholinergic, glutamatergic, and parvalbumin‐expressing GABAergic neurons in the basal forebrain were both active both during wakefulness and REM sleep.^[^ ^37^ ^]^ However, optogenetic activation of these neurons only promotes wakefulness from NREM sleep, but not from REM sleep. Considering that NREM and REM sleep have distinct EEG/EMG properties and are controlled by different neural circuits,^[^ ^13^, ^38^, ^39^ ^]^ these results suggest that TeA CaMKIIα+ neurons play a specific role in promoting the transition from NREM sleep to wakefulness transition. In addition, our results together with previous findings, suggest a high degree of specialization, rather than redundancy, among wakefulness‐promoting circuits in the mammalian brain.

### 3.2 Sound‐Evoked Wakefulness: Intensity and Sensitivity

The mechanism underlying sound‐evoked wakefulness is generally understood as a dynamic process in which louder sounds are more likely to induce wakefulness by exceeding perceptual thresholds, while quieter sounds may not, although several factors are involved, including the salience of the sound,^[^ ^17^ ^]^ the individual's sleep stage,^[^ ^18^ ^]^ and neural gating mechanisms that modulate sensory input during sleep.^[^ ^20^ ^]^ Previous studies involving the thalamus and brainstem have typically used relatively loud sounds (65–100 dB) to ensure a high probability of arousal.^[^ ^7^, ^8^ ^]^ However, as shown in Figure 2, we found that even soft sounds as low as 40 dB can still induce arousal, provided that the TeA is in a “high” state. Similar gating effects are observed at all intensity levels tested, from 40 to 80 dB, suggesting that the TeA likely plays a dynamic role in promoting arousal to softer sounds and preventing arousal from louder sounds by adjusting the brain's sensitivity to external stimuli. These findings also align with real‐world experiences, where soft whispers can sometimes wake an individual while louder sounds may not, suggesting that arousal thresholds are dynamic and that the TeA may play a pivotal role in this regulation. Compared to the mPFC(medial prefrontal cortex)^[^ ^40^ ^]^ and ACC(anterior cingulate cortex),^[^ ^41^, ^42^ ^]^ which are also cortical regions involved in sleep state regulation but not in sensory‐induced arousal, our findings offer new insights into how top‐down processes regulate sensory‐induced sleep‐wake transitions.

In addition, it is interesting to note that the low‐frequency oscillation we found by comparing the brain states associated with stronger and weaker neural responses matches well with the delta oscillation, which is often considered as the hallmark of deep sleep. This may also be related to the well‐known rule that waking up is easier during lighter sleep stages (N1 and N2) and more difficult during deep sleep (N3).^[^ ^43^ ^]^ Although it remains unclear why brain states have such a significant effect on the response levels of TeA neurons to the same sound, this may suggest a possible explanation for why individuals in deep sleep conditions have more difficulty waking up to external stimuli, which may be related to reduced response levels of TeA neurons. The low‐frequency delta oscillations may create a neural environment that either facilitates or inhibits the propagation of sensory signals necessary for wakefulness.

### 3.3 Functional Neural Pathways and Their Ecological Significance

Our investigation identified the TeA‐BLA/La glutamatergic pathway as the primary pathway for sound‐evoked arousal from NREM sleep. Activation of this pathway reliably induces wakefulness, underscoring its critical role in processing auditory information relevant to arousal. In contrast, although the TeA‐LPMR pathway can also lead to arousal upon optogenetic stimulation, it does not respond to 70 dB noise stimulation. This difference suggests that the BLA/La pathway has a specialized function in auditory arousal, whereas the LPMR pathway may be involved in other specific sleep‐wake regulation. In addition, these results also suggest that neurons in TeA may exhibit functional heterogeneity associated with projection specificity, consistent with previous studies that TeA functions as a center for multisensory processing.^[^ ^21^, ^23^ ^]^

The selective sensitivity of the TeA‐BLA/La pathway to varying sound intensities may have important ecological and behavioral implications. In natural environments, the ability to detect and respond to a range of auditory cues is essential for survival.^[^ ^44^ ^]^ For example, the detection of environmental sounds, such as the increasing rustling of leaves indicating the approach of a predator, requires increased sensitivity, which our results suggest could be facilitated by the TeA‐BLA/La pathway. Conversely, the ability to modulate responses to louder, potentially non‐threatening sounds prevents unnecessary awakenings that could disrupt restorative sleep. This selective gating ensures that organisms remain alert to critical auditory signals while maintaining uninterrupted sleep in the presence of benign environmental sounds. Furthermore, the ecological relevance of this pathway is evident in social contexts. For example, the role of the TeA in processing vocal communication, as demonstrated in maternal behavior,^[^ ^25^ ^]^ highlights its importance in socially relevant auditory processing. Such interesting open questions warrant further investigation.

### 3.4 Modulating Factors Influencing Sound‐Evoked Arousal

Besides the gating role of the TeA‐BLA/La pathway in sound‐evoked arousal, our results also indicate that the threshold for TeA‐driven arousal is not fixed, but may shift under certain conditions, creating the possibility for a nonlinear change in sensitivity to loudness during NREM sleep, as reflected by the intensity‐dependent threshold change shown in Figure 2G. This variable threshold raises the possibility of a multilevel gating mechanism, in which additional circuits or mechanisms beyond TeA may dynamically modulate the threshold at which TeA input elicits arousal. For example, BLA/La receives projections from TeA and is essential for sound‐evoked arousal, but BLA/La may also integrate input from other auditory or arousal‐related regions.^[^ ^45^ ^]^ Such convergent pathways could lower the threshold and thus reduce the amount of TeA activity needed to induce arousal, possibly through activation of subcortical regions at high sound pressure levels as previously reported,^[^ ^7^, ^8^, ^19^, ^20^ ^]^ or through neuromodulatory signals.^[^ ^20^, ^46^, ^47^, ^48^ ^]^ Although our study does not fully elucidate how these mechanisms interact, our results suggest that this multilevel gating mechanism is possible. Future investigations using targeted manipulations or additional tracking techniques could determine whether other brain regions actively modulate TeA‐driven arousal thresholds in different behavioral or physiological states.

It is also worth noting that there is emerging evidence that female sex hormones, particularly estrogen, have a significant impact on auditory processing. Fluctuations in estrogen levels during the estrous cycle have been shown to modulate the expression of estrogen receptors in the auditory system, thereby affecting auditory behaviors.^[^ ^49^ ^]^ In addition, estrogen has been found to have protective effects against acquired forms of hearing loss, underscoring its role in auditory function.^[^ ^50^ ^]^ Regarding the role of the TeA in maternal behavior, Tasaka et al. (2020) demonstrated that the TeA is critical for auditory‐driven maternal responses. Their study showed that TeA neurons show robust responses to ultrasonic vocalizations (USVs) of pups in maternal mice and that chemogenetic silencing of these neurons impairs pup retrieval behavior.^[^ ^25^ ^]^ This highlights the importance of the TeA in processing socially relevant auditory cues during maternity. Furthermore, research by Marlin et al. (2015) highlighted the role of oxytocin in modulating auditory cortical plasticity to facilitate maternal behavior. They found that oxytocin enables maternal responses to pup calls by balancing cortical inhibition, thereby enhancing the detection of pup USVs.^[^ ^51^ ^]^ This suggests that hormonal regulation is integral to the auditory processing adaptations observed in maternal mice.

### 3.5 Limitations and Concluding Remarks

While our study elucidates the pivotal role of TeA CaMKIIα+ neurons and their projections in sound‐evoked arousal from NREM sleep, several limitations warrant consideration. First, the anatomical proximity and overlapping projections between the TeA and the Ect, present challenges in distinctly attributing functional roles to each region. Although our retrograde labeling suggests potential contributions of both TeA and Ect, the specific involvement of Ect in sound‐evoked arousal remains speculative and requires further investigation using more precise targeting methodologies. In addition, our study exclusively utilized male mice to control for hormonal variability, which limits the generalizability of our findings across sexes. Future research incorporating female subjects and accounting for hormonal fluctuations is essential to fully understand the influence of sex‐specific factors on auditory processing and arousal mechanisms.

Remarkably, optogenetic activation of TeA neurons or their axon terminals within the TeA‐BLA/La pathway induces arousal from NREM sleep, even in the absence of sound stimuli. However, under normal conditions, TeA neurons appear to function only as gatekeepers for sound‐evoked arousal. This may be due to the artificial nature of optogenetic stimulation,^[^ ^52^, ^53^ ^]^ which bypasses natural gating and forces the TeA into a hyperactive state, making the brain extremely sensitive to sound and thereby inducing arousal by overactivating the BLA/La.^[^ ^54^ ^]^ This abnormality may help explain sleep disorders like insomnia, which are frequently linked to heightened sensitivity to external stimuli such as sound.^[^ ^55^ ^]^ Insights into the role of TeA in sound‐evoked arousal may guide the development of therapies for minimizing sound‐related sleep disturbances.^[^ ^56^ ^]^

## 4 Conclusion

Our study reveals that TeA CaMKIIα+ neurons, while not exhibiting significant activity changes during natural awakening from NREM sleep, play a pivotal role in modulating sound‐evoked arousal through the TeA‐BLA/La glutamatergic pathway. These findings uncover a gating mechanism by which the TeA regulates the brain's sensitivity to external auditory stimuli during sleep, advancing our understanding of sensory processing in sleep‐wake transitions. In addition, the specific role of the TeA also suggests that the neural pathways underlying sound‐evoked arousal from REM sleep should be different from TeA^Glut^‐BLA/La^Glut^.

## 5 Experimental Section

### Animals

Adult male C57BL/6J, Ai14 (Cre‐dependent tdTomato reporter, Jackson Laboratories, Stock No. 007909), and VGluT2‐IRES‐Cre (Jackson Laboratories, Stock No. 016963) mice aged 2–3 months were used in this study. The use of male mice allows us to focus on the mechanism without the potential influence of estrous cycle‐related hormonal fluctuations on sleep/wake states.^[^ ^25^ ^]^ All experimental procedures were approved by the Animal Care and Use Committee of the Army Medical University. All mice were housed under a 12‐h light‐dark cycle (lights on from 9 am to 9 pm) with ad libitum access to food and water.

### Sound Stimulation

All acoustic experiments were performed in a sound‐attenuating chamber where the background noise level was ≈20–25 dB SPL. The sinusoidal and noise signals were generated using a high‐speed DAQ (PCI‐6251, National Instruments, USA) with custom code written in Labview. The generated signals were then amplified (SA1, TDT Inc., USA) and output through an open‐field magnetic loudspeaker (MF1, TDT Inc., USA). For in vivo electrophysiological recordings, pure tones (10–80 dB SPL, 10 dB step; 2–32 kHz, 0.1 octave step; 35 ms duration; 5 ms sine ramp; 1000 ms interstimulus interval) were delivered for at least 10 repetitions in a pseudorandom order to obtain tonal receptive fields (TRFs). The generated sound was calibrated with a 1/4‐inch microphone (4135, Brüel and Kjaer, Denmark) for in vivo electrophysiological recording. The sound pressure level of white noise for sleep/wake study was calibrated using a sound pressure meter (SW‐524, Sndway, China).

### EEG/EMG Recording and Sleep/Wake States Detection

Mice were first anesthetized with isofluorane (60nL min^−1^). Two craniotomy holes were drilled above the frontal cortex (AP/ML: +1.5 mm/±1.5 mm) and two stainless steel screws were implanted as electrodes for EEG recording. For the reference electrode, the craniotomy hole was drilled at AP/ML: +2.5 mm/+2.5 mm of the left hemisphere over the parietal cortex. Similarly, two EMG electrodes were placed in the left and right trapezius muscles to monitor muscle activity. The EEG‐EMG electrodes were fixed to the skull with dental adhesive (Super‐Bond C&B, Sun Medical, Japan) and dental acrylic. After 5 days of recovery, the electrodes were connected to a data collector (OmniPlex, Plexon, USA or RM6240XC, Chengdu Instrument Factory, China) with flexible connecting cables, and mice were allowed to adapt for at least 3 days before recording.

EEG/EMG was recorded at a sampling rate of 1000 Hz and band‐pass filtered (EEG: 0.5–30 Hz, EMG: 30–200 Hz). A notch filter at 50 Hz was applied by the amplifier. Based on the EEG‐EMG waveforms and power spectra referenced in previous studies,^[^ ^57^, ^58^ ^]^ Three states were classified including NREM sleep, REM sleep, and wakefulness, using Sleepsign software and then manually corrected when necessary. A state of low‐amplitude EEG with low delta power (0.5–4 Hz) accompanied by random tonic EMG activity was classified as wakefulness, while a state of high‐amplitude EEG and high delta power accompanied by low‐amplitude EMG was classified as NREM sleep. A state of low‐amplitude EEG without EMG was classified as REM sleep if high theta waves (5–9 Hz) were obtained from the EEG power density. All states were categorized in consecutive, non‐overlapping 5‐s windows for each 5 s/epoch.

Two different types of latency to wakefulness were used in this study: latency to arousal and latency to wake. Latency to arousal was measured between stimulation onset and arousal indexed by the change in EEG, which was determined by an experimenter based on the absence of alpha oscillation, following previous research.^[^ ^26^ ^]^ For strong and fast arousal after optogenetic stimulation, latency to wake indexed by the change in EMG,^[^ ^7^ ^]^ was determined by an experienced researcher because the EEG change could be too short to be measured accurately.

### Sound‐Evoked Arousal Test

To minimize environmental noise, the sound‐evoked arousal test was conducted in a sound‐attenuating chamber where the background noise level was ≈20–25 dB SPL. The loudspeaker was placed 30 cm above the central floor of the box (length, 10 cm; width, 10 cm; height, 30 cm), and sound stimuli were delivered by a TDT System 3 (Tucker‐Davis Technologies, Alachua, USA). To study neural activity in response to auditory stimuli across the sleep‐wake state, white noise (40–80 dB SPL, 10 s duration) was applied in a pseudo‐random order with an inter‐stimulus interval (ISI) of 5–20 min (5–10 trials for each stimulus). To distinguish the neural responses corresponding to each brain state, sleep‐wake states were classified by the experimenter (J.C.W), who was blinded to the experimental conditions. According to the behavioral consequences of the sound stimuli, each trial was classified as NREM‐arousal or NREM‐unchanged. For NREM‐arousal trials, experimental mice should show at least 2 s of EEG desynchronization from the delta or theta rhythms, which is occasionally accompanied by a sudden increase in muscle activity for EMG within the time window of auditory stimulation. Otherwise, the trial was categorized as NREM unchanged.

### Virus Injecting

Surgery was performed using a stereotactic frame (RWD, Shenzhen, China) under isoflurane anesthesia. The body temperature of the animals was maintained at 36 °C with a heating pad throughout the procedure. Virus volumes ranging from 30 to 100 nL (depending on the desired expression level, viral titer, and target site size) were injected through calibrated glass micropipettes connected to an infusion pump (RWD, Shenzhen, China) at a rate of 60 nL min^−1^. After infusion, the pipette was left at the injection site for 10 min to prevent virus leakage from the injection site. Coordinates were determined based on three elements: anterior‐posterior (AP) distance from bregma, medial‐lateral (ML) distance from the midline, and dorsal‐ventral (DV) distance from the brain surface. Viral injections were performed at the following stereotaxic coordinates: TeA, AP: −2.8 mm, ML: ± 4.45 mm, and DV: 3.35 mm. The coordinates (AP/ML/DV) used for BLA/La were −1.2 mm, ± 3.1 mm, and 4.35 mm. Fiber optic ferrules were implanted at the following stereotaxic coordinates: TeA, AP −2.8 mm, ML ± 4.45 mm, and DV 3.1 mm; BLA/La, AP −1.2 mm, ML ± 3.1 mm, and DV 4.15 mm; LPMR, AP −2.2 mm, ML ± 1.75 mm, and DV 2.5 mm; AStr, AP −1. 2 mm, ML ± 3.1 mm, and DV 3.0 mm; VRe, AP −1.3 mm, ML ± 0.5 mm, and DV 4.2 mm; Cl, AP +1.1 mm, ML ± 3.2 mm, and DV 3.5 mm; LC, AP −5.6 mm, ML ± 1.0 mm, and DV 3.1 mm.

For tran‐synaptic anterograde tracing, AAV‐hSyn‐CRE‐WPREs (AAV2/1, 1.08 × 10^13^ vg mL^−1^, 50 nL, Brain VTA) were injected into the TeA of Ai14 mice to allow the virus to spread anterogradely to the downstream soma to express Cre and combined with the fluorescent protein td‐Tomato.^[^ ^59^ ^]^ For retrograde monosynaptic tracing, AAV‐hSyn‐CRE‐WPRE‐Hgh (AAV2/Retro, 5.22 × 10^12^ vg mL^−1^, 60 nL, Brain VTA) was injected into the BLA/La of Ai14 mice to allow the virus to spread retrogradely to the upstream soma to express Cre and combined with the fluorescent protein td‐Tomato. For calcium signal recording, rAAV‐CaMKIIa‐GCaMP6s‐WPRE‐hGH‐polyA (AAV2/9, 5.31 × 10^12^ vg mL^−1^, 50 nL) virus and rAAV‐CAG‐DIO‐axon‐jGCaMP7b‐WPRE‐hGH (AAV2/9, 4.65 × 10^12^ vg mL^−1^, 50nL) viruses were used. For optogenetic manipulation, rAAV‐CaMKIIa‐hChR2‐(H134R)‐mCherry‐WPRE‐polyA (AAV2/9, 5.31 × 10^12^ vg mL^−1^) and rAAV‐CaMKIIa‐eNpHR3.0‐mCherry‐WPRE‐hGH (AAV2/9, 5.28 × 10^12^ vg mL^−1^) were used. For chemogenetic manipulation, the pAAV‐CaMKIIa‐hM4D(Gi)‐mCherry‐3xFLAG‐WPRE (AAV2/9, 5.13 × 10^12^ vg mL^−1^, Obio Technology, Shanghai, China) and rAAV‐EF1α‐DIO‐hM4D(Gi)‐EYFP‐WPRE (AAV2/9, 5.4 × 10^12^ vg mL^−1^) viruses were used. In addition, rAAV‐CaMKIIa‐mCherry‐WPRE‐pA (AAV2/9, 5.21 × 10^12^ vg mL^−1^) and rAAV‐EF1α‐DIO‐EYFP‐WPRE (AAV2/9, 5.24 × 10^12^ vg mL^−1^) viruses were used as controls. A period of 3 weeks was allowed for virus expression, after which anatomical observation, functional recording, or optogenetic/chemogenetic manipulation was conducted. Unless otherwise noted, the viruses used in this study were provided by Brain VTA Co. (Wuhan, China).

### Optogenetic Manipulation

For somatic optogenetic activation, rAAV2/9‐CaMKII‐hChR2(H134R)‐mCherry (5.31 × 10^12^ vg mL^−1^) was randomly injected into TeA in one hemisphere of C57BL/6J mice. For somatic optogenetic inhibition, rAAV2/9‐CaMKII‐eNPHR3.0‐mCherry (60 nL) was injected into both sides of TeA. The optical fiber cable (200 µm diameter) was connected to a 473 nm blue or 589 nm yellow laser (Aurora‐200, Newdoon, Hangzhou, China) via an FC/PC adaptor, and the light pulses were controlled by a multichannel signal generator (Master 9, AMPI, Israel). The fiber optic cable and EEG‐EMG electrodes were connected to the implanted fiber optic ferrule and connectors 2 h before each experiment. The laser power at the tip of the optical fiber was measured and calibrated using a light power meter (PM100D, Thorlabs, USA). For optogenetic activation during sleep, different laser pulses (1 Hz, 5 Hz, 10 Hz and 20 Hz, 5 mW, 15 ms per pulse, 10 s) were applied every 5–20 min. For optogenetic inhibition during sleep, continuous yellow laser (10 mW, 60 s) was delivered during stable NREM sleep (at least 20 s of continuous NREM sleep) every 5–20 min. For optogenetic activation of projecting terminals from TeA, laser pulses (10 Hz, 10 mW, 15 ms per pulse, 10 s) were applied. To minimize injury to the cortex, optic fibers were positioned vertically on the surface of the dura mater of the TeA (posteriorly 2.8 mm from Bregma, laterally 0.15 mm from the rhinal fissure). Each experimental session lasted for 1–3 h.

### Chemogenetic Manipulations

For chemogenetic inhibition of TeA CaMKII+ neurons, C57BL/6J mice were bilaterally injected with rAAV2/9‐CaMKII‐hM4D(Gi)‐mCherry or rAAV2/9‐CaMKII‐mCherry (control) in a volume of 60 nL. For circuitry inhibition, C57BL/6J mice received bilateral injections of retroAAV‐hSyn‐cre virus into the BLA/La (80 nL), followed by an injection into the TeA (50 nL) with either rAAV2/9‐EF1a‐DIO‐hM4Di‐eYFP or AAV2‐EF1a‐DIO‐eYFP (control). EEG‐EMG electrodes were subsequently implanted. Three weeks later, injected mice were connected to the data acquisition system for EEG and EMG recording. Mice were habituated for 3 days prior to injection and EEG‐EMG recording.

For chemogenetic modulation of TeA CaMKII+ neurons, CNO or saline was administered 30 min before recording at 21:00 (onset of dark phase). For circuitry inhibition, CNO or saline was administered during the day (duration of light phase) and recordings were made 30 min after injection and continued for 3 h. An intraperitoneal injection of clozapine‐N‐oxide (CNO, 5 mg kg^−1^, Sigma, USA) or saline was administered 30 min prior to behavioral testing. Mice were allowed to recover from either CNO or saline injection for at least 3 days before the next experiment.

### Fiber Photometry in Freely Behaving Mice

Calcium activity recordings were conducted using a fiber photometry system (C1410488, Inper, China). An optical fiber ferrule (200 µm diameter) was slowly implanted into the target areas, 3 weeks after AAV injection. Mice were allowed to recover for 7 days. Activity‐dependent calcium fluorescence signals (470 nm) and activity‐independent fluorescence signals (405 nm) were captured using a CMOS camera, providing an internal control for motion and bleaching artifacts. Before data analysis, baseline correction was performed using Inper Data Processing (Inper, China) or Matlab (MathWorks, USA). For the calculation of ΔF/F, the baseline F0 was defined as the average fluorescence signal (F) during the 10 s before the onset of sound stimuli or the transition from sleep to wake.

### In Vitro Electrophysiological Recording in the TeA or BLA/La

Mice were deeply anesthetized with sodium pentobarbital (60–70 mg kg^−1^) and then decapitated. After rapid removal of the mouse brain, 300 µm coronal slices containing the TeA or BLA/La from the virus‐infected hemisphere were prepared in ice‐cold artificial cerebrospinal fluid (ACSF) (in mM: 124 NaCl, 2.5 KCl, 1.2 NaH_2_PO_4_, 25 NaHCO_3_, 1 MgCl_2_, 2 CaCl_2_, 20 glucose; 295 mOsm; pH 7.3) using a vibratome (VT1200S, Leica, Germany). The slices were transferred to the holding chamber and incubated in ACSF at 30 °C for 30 min for recovery. Prior to recording, the slices were kept at room temperature and continuously bubbled with 95% O_2_ and 5% CO_2_. The slices were then immersed in a recording chamber and perfused with oxygenated ACSF at a rate of 3–4 mL min^−1^. Whole‐cell patch‐clamp recordings were conducted under an upright microscope (Leica DM6 FS, Germany), which was equipped with interference contrast (IR/DIC) and an infrared camera. Patch pipettes were pulled from borosilicate glass capillaries (outer diameter of 1.5 mm, Vital Sense, Germany) using a horizontal puller (P97, Sutter Instrument, USA). Patch pipettes were filled with a potassium‐based internal solution containing 140 mM K+‐gluconate, 9 mM HEPES, 5 mM EGTA, 4 mM Mg‐ATP, 0.3 mM GTP, 4.5 mM MgCl_2_, and 4.4 mM phosphocreatine sodium. The pH was adjusted to 7.3 with cesium hydroxide, and the osmotic pressure was ≈295 mOsm. The signals were acquired after low‐pass filtering at 2.8 kHz and digitized at 10 kHz via a patch clamp amplifier (Multiclamp 700B, Molecular Devices, USA). The laser was delivered through an optical fiber (200 µm diameter, Newdoon, China) positioned 0.2 mm above the surface of the target brain region in the brain slice.

To examine the action potential fidelity of CaMKIIα+ neurons in the TeA to optogenetic stimulations, AAV‐CaMKIIa‐hChR2(H134R)‐mCherry was injected into the TeA. After 3 weeks, mCherry‐labeled neurons expressing ChR2 in the TeA were recorded under visual guidance using a fluorescent microscope. The action potential of the target neuron in response to laser stimulation (473 nm, 10 mW, 15 ms) at different frequencies (1, 5, 10, 15, and 20 Hz) was recorded in I‐clamp mode.

To study the synaptic input from TeA CaMKIIα+ neurons to BLA/La glutamatergic neurons, AAV‐CaMKIIa‐hChR2(H134R)‐eYFP was injected into the TeA and AAV‐DIO‐mCherry was injected into the BLA/La of VGluT2‐IRES‐Cre mice. Excitatory postsynaptic potentials including action potentials (EPSPs) of mCherry‐labeled neurons in the BLA/La were recorded in current clamp mode in response to blue laser stimulation at different frequencies (1, 10, and 20 Hz; 473 nm, 10 mW, 15 ms). In addition, laser‐evoked excitatory postsynaptic currents (EPSCs) were recorded in voltage clamp mode by clamping the membrane potential at −70 mV.

### In Vivo Electrophysiological Recording in Head‐Fixed Mice

Mice were first anesthetized with sodium pentobarbital (60–70 mg kg^−1^) and then the head was fixed in a stereotaxic apparatus (RWD, Shenzhen, China). After the scalp was removed, a reference electrode was placed in a hole drilled above the frontal cortex to make good contact with the cerebrospinal fluid. A customized nail was then glued to the cranial surface above frontal cortex with dental cement for fixation. A cranial window was then opened above the TeA (AP: −2.4 to −3.2 mm, ML: lateral ≈0.15 mm from the rhinal fissure) without removing the dura. The exposed brain was covered with Vaseline to prevent dehydration. During the TeA recording, the animals head was rotated 80° laterally to facilitate access to the recording site. After surgery, local anesthetic (lidocaine hydrochloride) and antibiotic ointment (erythromycin) were applied to the surgical wound, and each mouse was housed alone for at least 5 days to recover.

For in vivo loose patch clamp recording, the awake mouse was head‐mounted on a holder. Vaseline and Dura were removed. Loose‐patch recordings were performed using a similar configuration as for in vitro electrophysiological recordings, except that the glass pipette (tip: 1.0 mm in diameter, 5–7 MΩ impedance) was filled with ACSF in mM: 124 NaCl, 2.5 KCl, 1.2 NaH_2_PO_4_, 25 NaHCO_3_, 1 MgCl_2_, 2 CaCl_2_, 20 glucose, 1% biocytin (pH 7.3, 295 mOsm). Technical details of the operation could be found in previous reports.^[^ ^60^ ^]^ The recorded signals were bandpass filtered at 300–3000 Hz to detect spikes activities. After data acquisition, the cell was stimulated with low‐intensity positive current pulses (I = 5 pA) for 2 min in current‐clamp mode to facilitate membrane rupture and label the neuron with biocytin.^[^ ^61^ ^]^ At the end of the experiment, the mice were anesthetized with sodium pentobarbital and then transcardially perfused in preparation for biocytin staining to confirm that the recorded neurons were in the TeA.

### Immunohistochemistry and Fluorescent Imaging

Mice were deeply anesthetized with an intraperitoneal injection of pentobarbital sodium and subsequently perfused with ice‐cold 0.9% saline followed by 4% PFA. The brains were then extracted and placed in 4% PFA (prepared in PBS) at 4 °C overnight, followed by incubation in 20% sucrose until they sank. Coronal slices of 35 µm thickness were prepared using a cryostat (CM1860, Leica, Germany).

For immunofluorescence, the sections were incubated in a blocking buffer (1% Triton X‐100, 10% donkey serum in PBS) for 1 h at room temperature. Subsequently, they were treated with primary antibodies diluted with blocking solution, including anti‐glutamate (1:500, rabbit, G6642, Sigma, USA), anti‐GABA (1:1000, rabbit, A2052, Sigma, USA), and anti‐c‐Fos (1:800, rabbit, ab190289, Abcam, UK), at 4 °C for 24 h. For biocytin staining, sections were incubated with streptavidin‐Cy3 (1:200, catalog no. 43–4315, Molecular Probes, USA). After three washes with PBS, the slices were incubated with the corresponding fluorophore‐conjugated secondary antibodies (goat anti‐rabbit 488, 1:500, ab150077, Abcam, UK) for 2 h at room temperature. Following additional rinsing steps, the slices were incubated in 4,6‐diamidino‐2‐phenylindole (DAPI; 1:2000, Sigma, USA) for 5 min at room temperature. The slices were then scanned and imaged using a fluorescence microscope (VS200, Olympus, Japan) or a confocal laser scanning microscope (Spin10, Olympus, Japan) to visualize the fluorescence signals.

For c‐Fos staining, mice were randomly divided into two groups: a control group without sound stimulation, and an experimental group exposed to 70 dB SPL white noise for 90 min. Since c‐Fos protein expression has been shown to peak at 60–180 min after stimulus onset,^[^ ^62^ ^]^ pentobarbital‐anesthetized mice were chosen to perfuse for 90 min after the onset of noise stimulation.

### Statistical Analysis

All data are expressed as mean ± SEM unless otherwise specified. NeuroExplorer 5 (Plexon, USA) and SleepSign (Kissei Comtec, Japan) were used to analyze sleep status. Statistical analysis was performed with GraphPad Prism 8 (Dotmatics, USA) or Matlab (MathWorks, USA). Two‐tailed paired t‐test, two‐tailed Unpaired t‐test were used to test for statistical significance. All statistics passed the D'Agostino‐Pearson test and met the prerequisites of the parametric testing. P values were adjusted using the Tukey method with a significance level of 0.05 (n.s., *p* > 0.05; *, *p* < 0.05; **, *p* < 0.01; ***, *p* < 0.001; ****, *p* < 0.0001).

## Conflict of Interest

The authors declare no conflict of interest.

## Author Contributions

Conceptualization was carried out by H.P.Y, J.C.W, W.Y, H.X, Z.C, B.H.L, X.W.C, S.C.R, and Y.Z. The methodology was developed by H.P.Y, R.Q.P, P.H.C, B.M.L, T.T.L, C.H, X.Z, Y.T.L, and Z.Y.S. The investigation was conducted by H.P.Y, J.C.W, R.Q.P, P.H.C, M.Q.G, M.Y.L, Y.T.Z, Q.Q.W, and B.M.L. Visualization efforts were led by J.C.W, H.P.Y, and Y.Z. Funding acquisition was managed by S.C.R, X.W.C, and Y.Z. Project administration was handled by S.C.R, X.W.C, and Y.Z. Supervision was provided by Y.Z. The original draft was written by H.P.Y, J.C.W, R.Q.P, and Y.Z, while the writing — review and editing were performed by H.P.Y, J.C.W, S.C.R, X.W.C, and Y.Z.

## Supporting information



Supporting Information

## Data Availability

The data that support the findings of this study are available from the corresponding authors upon reasonable request.
