# Supplementary material for: Temporal Association Cortex Gates Sound‐Evoked Arousal from NREM Sleep
Source: Adv Sci (Weinh). 2025 Jan 31;12(12):2414271. doi: 10.1002/advs.202414271 (PMC11948000; doi:10.1002/advs.202414271)
Supplement: Supplementary file 1 — Supporting Information [file ADVS-12-2414271-s001.docx]

Supporting Information

Temporal Association Cortex Gates Sound-Evoked Arousal From NREM Sleep

Haipeng Yu^*^, Jincheng Wang^*^, Ruiqi Pang^*^, Penghui Chen, Tiantian Luo, Xuan Zhang, Yatao Liao, Chao Hu, Miaoqing Gu, Bingmin Luo, Zhiyue Shi, Mengyao Li, Yueting Zhang, Qiaoqian Wei, Wei Yuan, Hui Xie, Zhiyi Chen, Hongbang Liu, Shuancheng Ren**^#^**, Xiaowei Chen**^#^**, Yi Zhou**^#^**


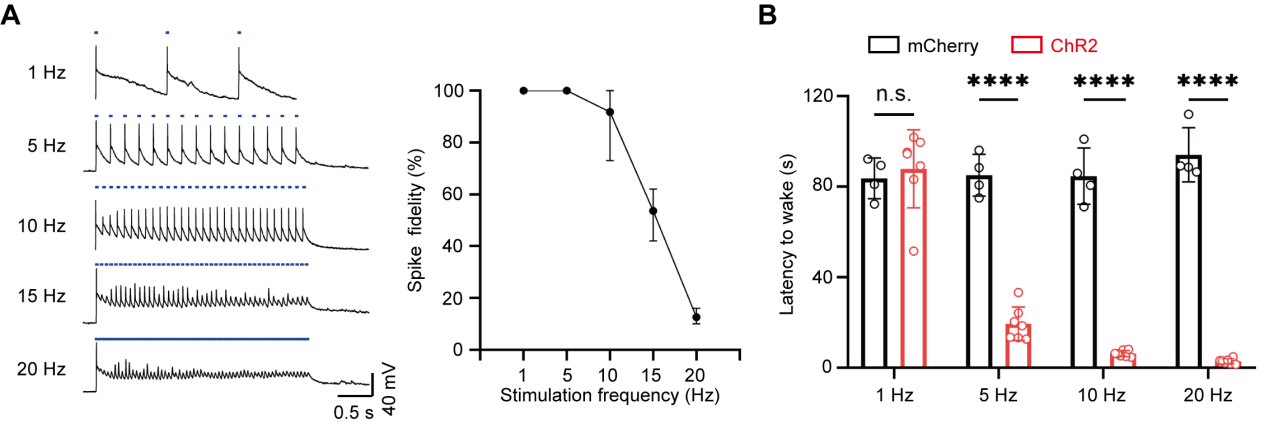


**Figure. S1. Functional verification of ChR2 activation in TeA CaMKIIα+ neurons.**

1. Left: Representative EPSCs in response to different frequencies of blue light (473 nm) stimulation. Right: Firing fidelity of ChR2-expressing TeA neurons to different blue light stimulation frequencies, n = 6 cells. Data are represented as mean ± range. (B) Latency to wake from NREM sleep after optical activation at 1 Hz, 5 Hz, 10 Hz, or 20 Hz. ChR2, n = 7 mice; mCherry, n = 4 mice. ****, P < 0.0001; n.s., not significant. Error bars represent SEM. See Table S1 for details on statistical data analysis.


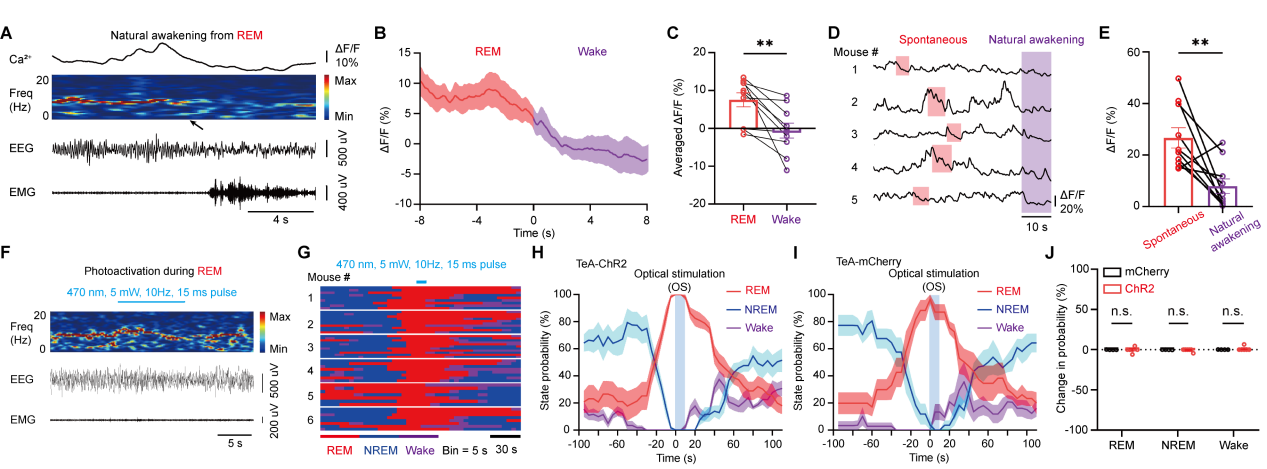


**Figure. S2. Role of TeA CaMKIIα+ neurons in the transition from REM sleep to wake.**

1. Example of REM-wake transition. (B) Averaged Ca^2+^ activity during the REM-wake transition, 10 transitions from 6 mice. (C) Comparison of averaged ΔF/F values within 8s before and after the transition, n = 6 mice. (D) Five representative traces containing Ca^2+^ activity before and after natural awakening. Purple shading indicates the natural awakening. (E) Comparison of spontaneous activity (red shading in D) during REM sleep with the change in amplitude of Ca^2+^ activity during natural awakening from REM sleep. The amplitude of spontaneous activity (manually picked) was calculated from the difference between the peak amplitude and the lowest activity within 10 seconds following the peak. The amplitude change of Ca^2+^ activity during natural awakening from REM sleep was calculated as the difference in averaged activity 8 seconds before and after arousal. (F) Representative sleep-wake state after 10Hz optogenetic stimulation during NREM sleep. (G) Photoactivation results from all trials in ChR2-expressing mice. (H) (I) Probability of each state around optogenetic stimulation during NREM sleep in the ChR2 group (H, n = 6 mice) and mCherry group (I, n = 4 mice). Rectangular shading represents the optogenetic stimulation (OS). (J) Probability change of each state 10s before and after stimulation onset during REM sleep. **, P < 0.01; ***, P < 0.001; n.s., not significant. Error bars represent SEM. See Table S1 for details on statistical data analysis.


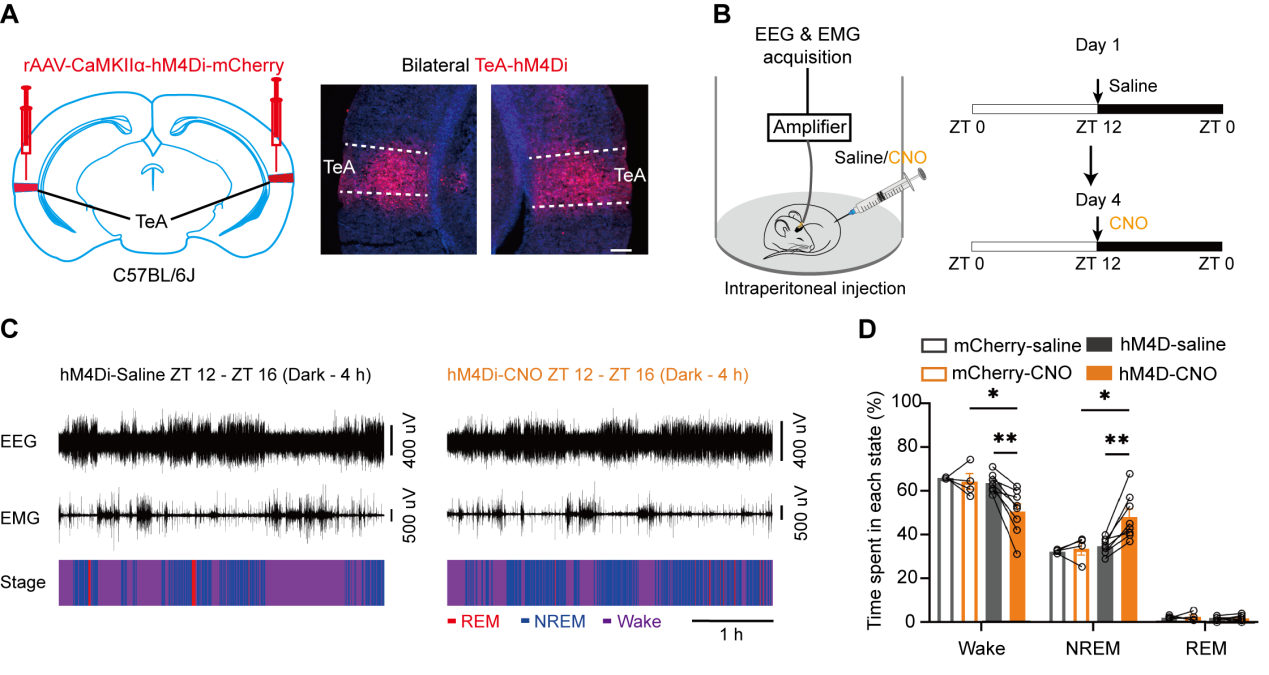


**Figure. S3. Chemogenetic inhibition of TeA CaMKIIα+ neurons promotes NREM sleep.**

1. Left:Schematic of virus injection in the TeA. Right: Representative expression of hM4Di-mCherry in the TeA, bilateral. Scale bar, 200 µm. (B) Left: EEG/EMG recording after intraperitoneal saline/CNO injection in freely moving mice. Right: Protocol for the delivery of saline or CNO. ZT: zeitgeber time. (C) Representative sleep/wake states for 4 hours in a hM4D-mCherry mouse after saline (left) or CNO (right) injection. (D) Percentage of time spent in each sleep/wake state during 4 hours after injection. hM4D group, n = 8 mice; mCherry group, n = 4 mice. *, P < 0.05; **, P < 0.01; n.s., not significant. Error bars represent SEM. See Table S1 for details on statistical data analysis.


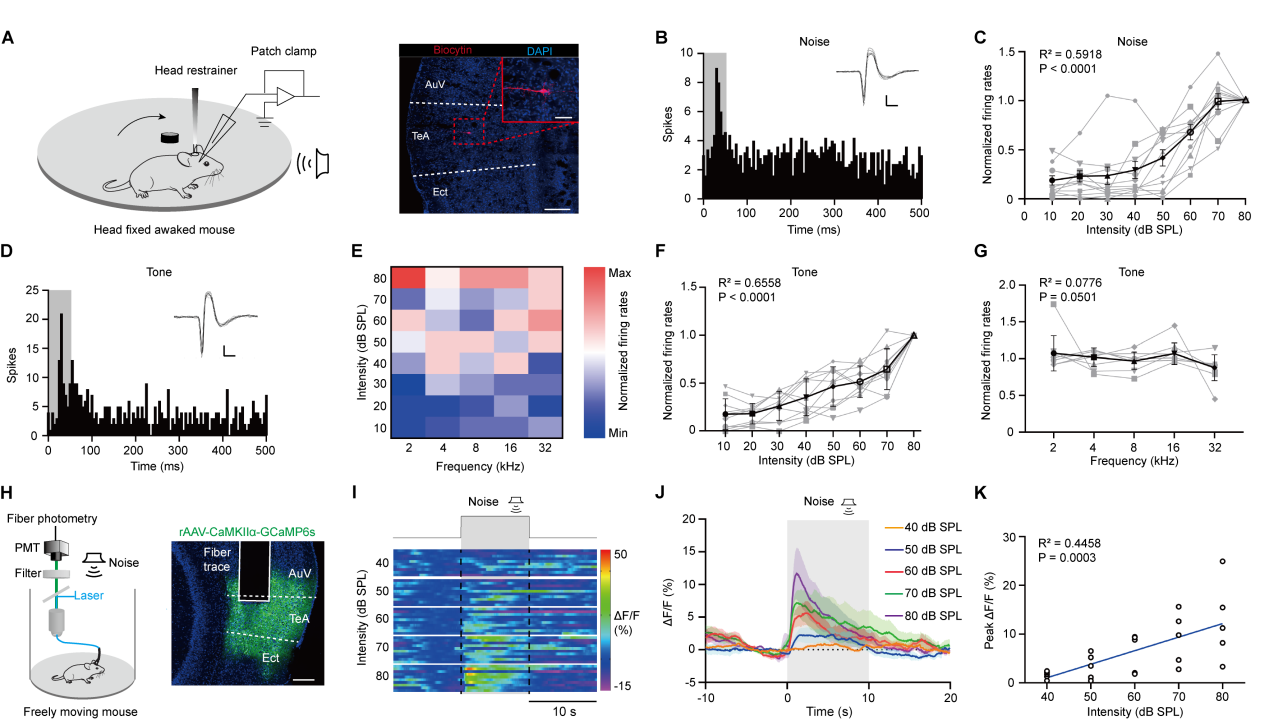


**Figure. S4. Response properties of TeA neurons to sound stimulus.**

1. Left: Schematic of in vivo patch clamp recordings. Right: Representative morphology of recorded neuron reconstructed by biocytin the TeA. Scale bar, 200 µm; inset scale bar, 50 µm. (B) Representative post-stimulus time histogram (PSTH) of a recorded TeA neuron responding to white noise with the spike waveform shown as an inset. Gray shading represents white noise. Scale bar: 50 mV, 0.8 ms. (C) Normalized firing rates evoked by noise at different intensity levels for all noise responsive neurons (n = 11). (D) Representative post-stimulus time histogram (PSTH) of a recorded TeA neuron responding to pure tones with the spike waveform shown as an inset. (E) The tonal receptive field (TRF) of the same neuron shown in D. (F) (G) Normalized firing rates evoked by pure tones at different intensity levels (F) and frequencies (G) for all tone responsive neurons (n = 10). (H) Left: Schematic of monitoring of TeA CaMKIIα+ neuron Ca^2+^ activity to noise stimulation in freely moving mice. Right: GCaMP6s expression and fiber trace in TeA. Scale bar, 200 µm. (I) Representative examples of TeA neurons responding to noise at different sound pressure levels. Dotted lines indicate the onset and offset of sound. (J) (K) Averaged TeA Ca^2+^ activity (J) and amplitude (K) evoked by different sound levels. n = 5 mice. Gray shading represents white noise. Error bars represent SEM. See Table S1 for details on statistical data analysis.


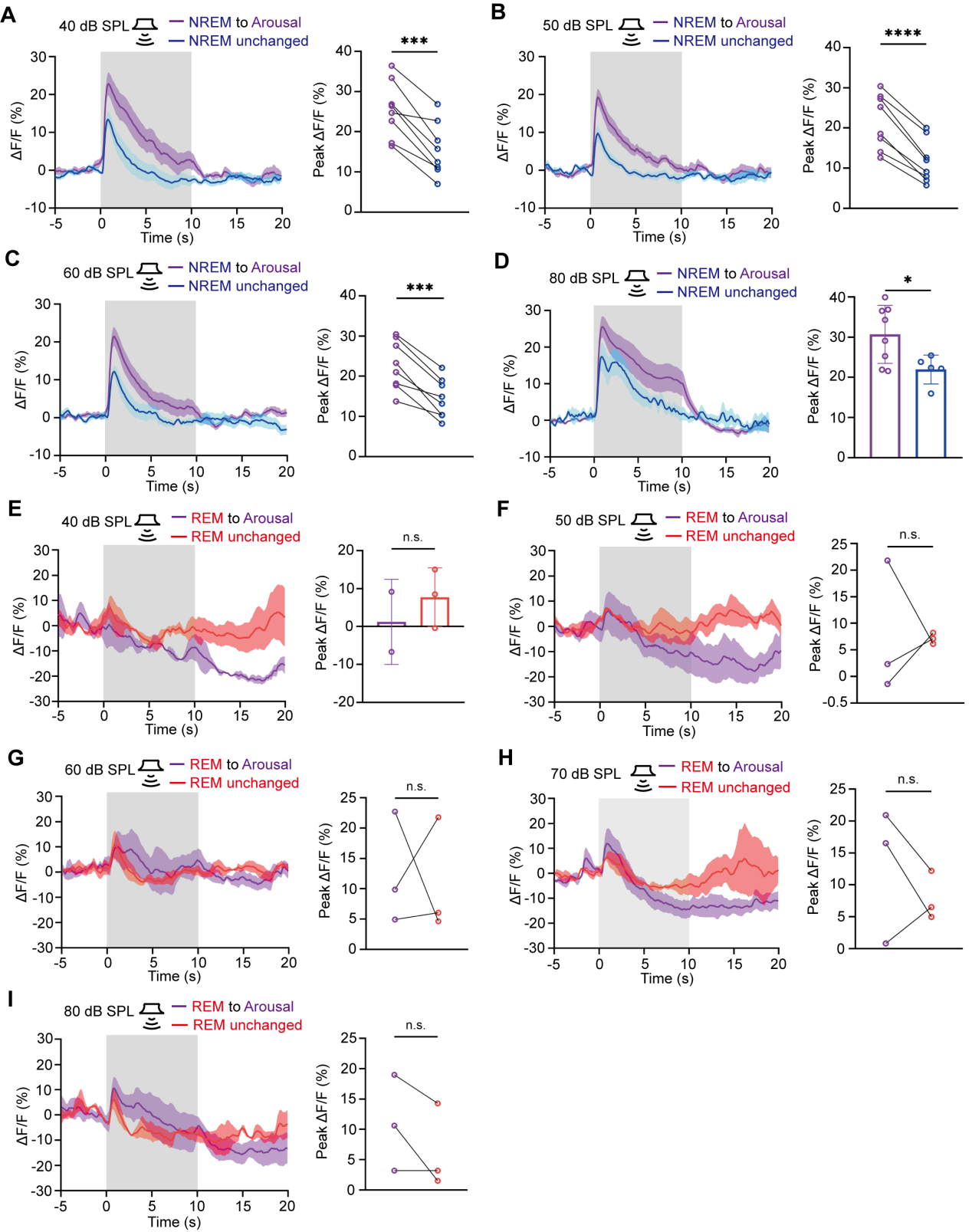


**Figure. S5.** **Evoked responses of TeA CaMKIIα+ neurons to different levels of noise during NREM and REM sleep**

1. (B) (C) (D) represent averaged sound-evoked Ca^2+^ activity of TeA CaMKIIα+ neurons during NREM sleep, in both aroused and non-aroused cases, when exposed to 40 dB SPL (A), 50 dB SPL (B), 60 dB SPL (C), and 80 dB SPL (D) noise, n = 8 mice. Response to 70 dB SPL noise has been shown in Figure. 2C. (E) (F) (G) (H) (I) represent averaged sound-evoked Ca^2+^ activity of TeA CaMKIIα+ neurons during REM sleep, in both aroused and non-aroused cases, when exposed to 40 dB SPL (E), 50 dB SPL (F), 60 dB SPL (G), 70 dB SPL (H), and 80 dB SPL (I), n = 3 mice. Gray shading represents the noise. A paired *t-test* was used whenever applicable. An Unpaired *t-test* was employed when arousal consistently occurred in response to loud sound, as no comparison could be made between arousal and non-arousal conditions. *, *P* < 0.05; ***, *P* < 0.001; n.s., not significant. Error bars represent SEM. See Table S1 for details on statistical data analysis.


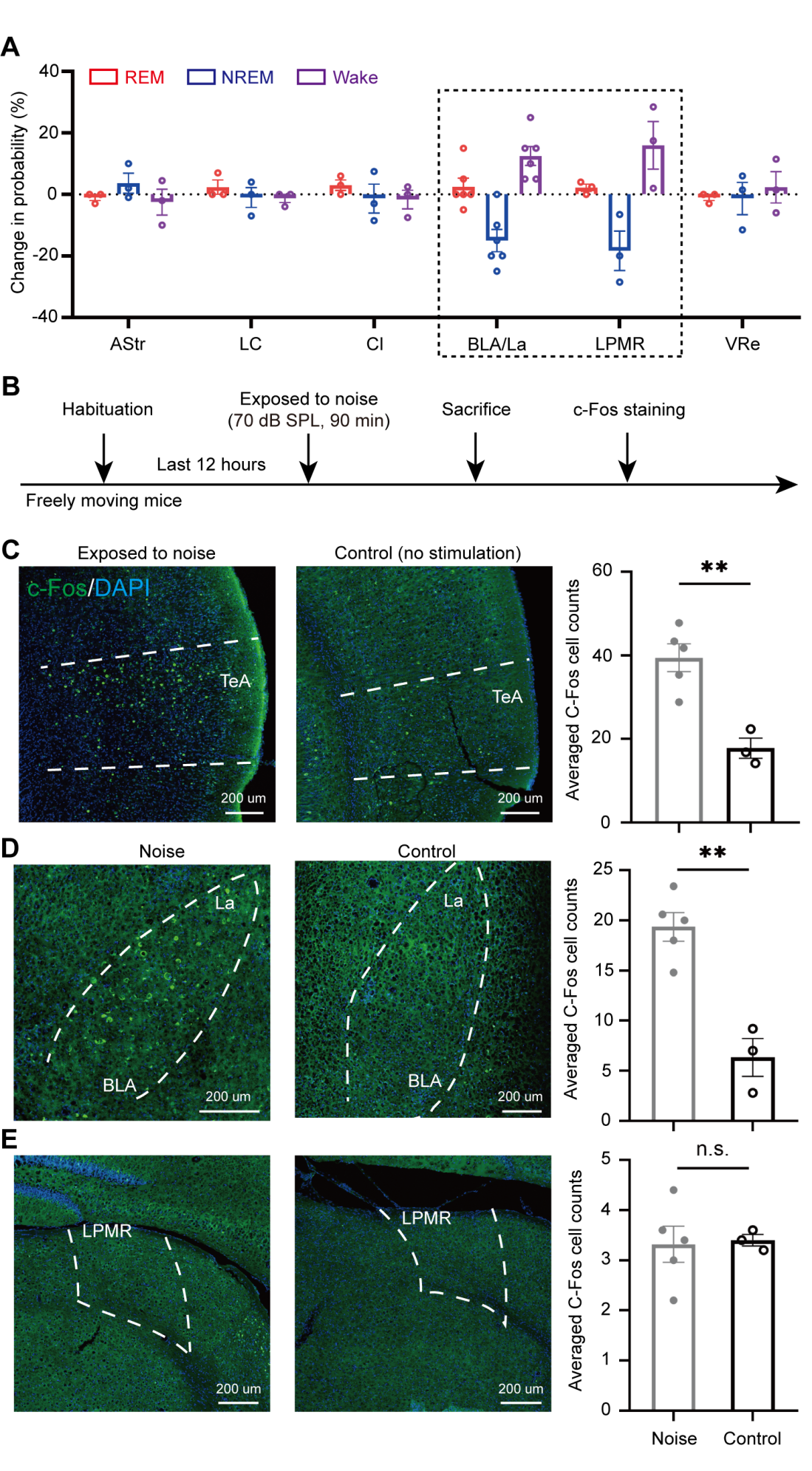


**Figure. S6. c-Fos expression of TeA, BLA/La and LPMR after noise exposure in freely moving mice**

1. Probability change of sleep/wake state during optogenetic stimulation in different downstream targets of TeA CaMKIIα+ neurons. Dashed line box indicates the down targets of TeA regulating sleep-wake transition. (B) Schematic of the experimental design. (C) (D) (E) c-Fos expression and cell counts in TeA, BLA/La and LPMR, respectively. Left: representative images of c-Fos expression after noise exposure; Middle: representative images of c-Fos expression without noise exposure (control); Right: the cell counts of c-Fos positive neurons. n = 5 mice for experimental group, n = 3 mice for control group. **p<0.01. n.s., not significant. Error bars represent SEM. See Table S1 for details on statistical data analysis.


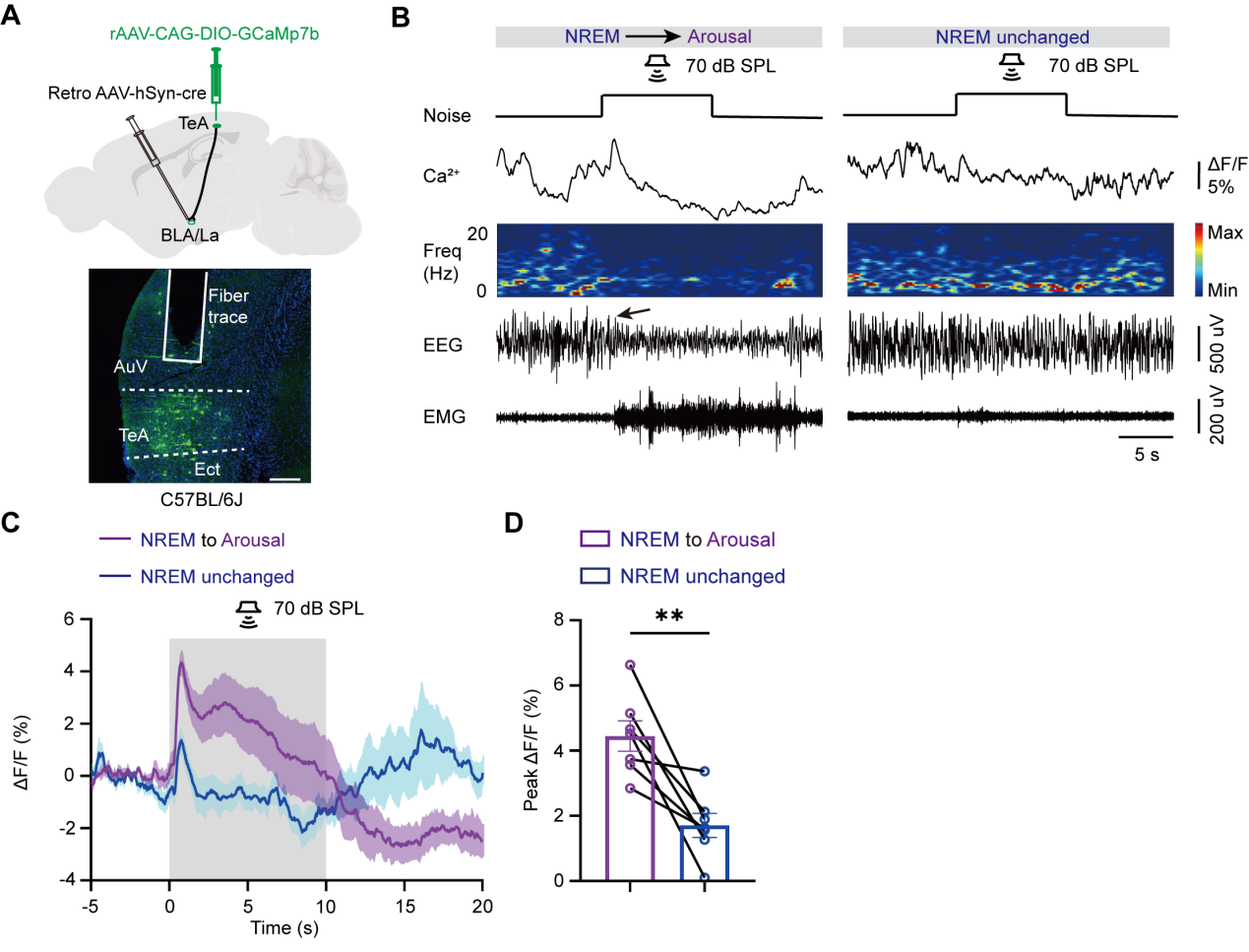


**Figure. S7. Ca^2+^ activity of TeA neurons projecting to BLA/La to 70 dB SPL noise during NREM sleep.**

1. Schematic of virus injection into the TeA and BLA/La for calcium recording with projection specificity. Scale bar, 200 µm. (B) Representative case where 70 dB SPL noise can evoke arousal (left) and cannot evoke arousal (right). Black arrow indicates the onset of arousal indexed by EEG change. (C) Averaged sound-evoked Ca^2+^ activity of TeA neurons projecting to BLA/La during NREM sleep, in both aroused and non-aroused cases. (D) Comparison of sound-evoked Ca^2+^ activities in aroused and non-aroused cases. n = 7 mice. **, *P* < 0.01. Error bars represent SEM. See Table S1 for details on statistical data analysis.

**Table S1. Summary of statistics**

| **Figure** | **Parameter** | **Groups** | **Number of samples** | **Statistics** | **Test** |
| --- | --- | --- | --- | --- | --- |
| Figure. 1G | Averaged ΔF/F in NREM and Wake | NREM/Wake | n = 22 traces from 6 mice | P = 0.3338 | Paired *t-test* |
| Figure. 1M | State change in probability | NREM, REM and Wake | NREM to Wake: n = 8 mice;  REM to Wake: n = 6 mice | REM: P = 08337;  NREM: P < 0.0001;  Wake: P < 0.0001 | Unpaired *t-test* |
| Figure. 1N | Latency from laser on to Wake | NREM/REM | NREM to Wake: n = 7 mice;  REM to Wake: n = 6 mice | P < 0.0001 | Unpaired *t-test* |
| Figure. 2D | Peak ΔF/F in NREM to Arousal and NREM unchanged trials | NREM to arousal; NREM unchanged | n = 8 mice | P = 0.0119 | Paired *t-test* |
| Figure. 2F  c | Sound-evoked Ca^2+^ activity | 40dB SPL; 50dB SPL; 60dB SPL; 70dB SPL; 80dB SPL | n = 8 mice | 0dB SPL, mean = 19.04 %; 50dB SPL, mean = 15.83 %; 60dB SPL, mean = 18.74 %; 70dB SPL, mean = 21.76 %; 80dB SPL, mean = 29.49 % | Descriptive statistics |
| Figure. 2G | Sound-evoked Ca^2+^ activity and whether arousal | 40dB SPL; 50dB SPL; 60dB SPL; 70dB SPL; 80dB SPL | n = 8 mice | 40dB SPL, Tjur's R^2^ = 0.2740; 50dB SPL, Tjur's R^2^ = 0.1531; 60dB SPL, Tjur's R^2^ = 0.2262; 70dB SPL, Tjur's R^2^ = 0.1477; 80dB SPL, Tjur's R^2^ = 0.0804 | Simple logistic regression |
| Figure. 2L | Sound-evoked arousal probability | Laser on in mCherry group; Laser off in mCherry group; Laser on in eNpHR3.0 group; Laser off in eNpHR3.0 group; | mCherry group: n = 4 mice;  eNpHR3.0 group: n = 6 mice | Laser on in mCherry group vs eNpHR3.0 group: P = 0.0020.  Laser on vs Laser off in eNpHR3.0 group: P = 0.0003 | Laser on in mCherry group vs eNpHR3.0 group: Unpaired *t-test*.  Laser on vs Laser off in eNpHR3.0 group: Paired *t-test* |
| Figure. 2M | Latency to sound-evoked arousal | Laser on in mCherry group; Laser off in mCherry group; Laser on in eNpHR3.0 group; Laser off in eNpHR3.0 group; | mCherry group: n = 4 mice;  eNpHR3.0 group: n = 6 mice | Laser on in mCherry group vs eNpHR3.0 group: P = 0.0207.  Laser on vs Laser off in eNpHR3.0 group: P = 0.0217 | Laser on in mCherry group vs eNpHR3.0 group: Unpaired *t-test*.  Laser on vs Laser off in eNpHR3.0 group: Paired *t-test* |
| Figure. 2N | Duration of sound-evoked arousal | Laser on in mCherry group; Laser off in mCherry group; Laser on in eNpHR3.0 group; Laser off in eNpHR3.0 group; | mCherry group: n = 4 mice;  eNpHR3.0 group: n = 6 mice | Laser on in mCherry group vs eNpHR3.0 group: P < 0.0001.  Laser on vs Laser off in eNpHR3.0 group: P = 0.0116 | Laser on in mCherry group vs eNpHR3.0 group: Unpaired *t-test*.  Laser on vs Laser off in eNpHR3.0 group: Paired *t-test* |
| Figure. 3B | Averaged ΔF/F of baseline activity at sound onset in different sound level | 40dB SPL, 50dB SPL, 60dB SPL, 70dB SPL, 80dB SPL for NREM to arousal and NREM-unchanged trials, respectively. | n = 8 mice for 40dB, 50dB, 60dB, 70dB, 80dB, respectively | 40dB SPL: P = 0.0460; 50dB SPL: P = 0.0108; 60dB SPL: P = 0.4673; 70dB SPL: P = 0.4931; 80dB SPL: P = 0.6212 | 40dB, 50dB, 60dB, 70dB:Paired *t-test;*  80dB: Unpaired *t-test* |
| Figure. 3D | Normalized power of EEG signal in 2-seconds window before noise | Lowest calcium trials and highest calcium trials | n = 8 mice | 1.46-1.95 Hz: P = 0.3675 1.95-2.44 Hz: P = 0.0452 2.44-2.92 Hz: P = 0.0359  2.92-3.41 Hz: P = 0.0486  3.41-3.90 Hz: P = 0.1513 | Paired *t-test* |
| Figure. 6D | Probability of sound-evoked arousal | Saline injection in eYFP group; CNO injection in eYFP group; Saline injection in hM4D group; CNO injection in hM4D group; | eYFP group: n = 4; hM4D group: n = 6 | Saline vs CNO injection in hM4D group: P = 0.0026.  CNO injection in eYFP group vs hM4D group: P = 0.0012 | Saline vs CNO injection in hM4D group:Paired *t-test*.  CNO injection in eYFP group vs hM4D group:Unpaired *t-test* |
| Figure. 6E | Latency to sound-evoked arousal | Saline injection in eYFP group; CNO injection in eYFP group; Saline injection in hM4D group; CNO injection in hM4D group; | eYFP group: n = 4; hM4D group: n = 6 | Saline vs CNO injection in hM4D group: P = 0.0003.  CNO injection in eYFP group vs hM4D group: P < 0.0001 | Saline vs CNO injection in hM4D group:Paired *t-test*.  CNO injection in eYFP group vs hM4D group:Unpaired *t-test* |
| Figure. 6F | Duration of sound-evoked arousal | Saline injection in eYFP group; CNO injection in eYFP group; Saline injection in hM4D group; CNO injection in hM4D group; | eYFP group: n = 4; hM4D group: n = 6 | Saline vs CNO injection in hM4D group: P = 0.0397.  CNO injection in eYFP group vs hM4D group: P = 0.0229 | Saline vs CNO injection in hM4D group:Paired *t-test*.  CNO injection in eYFP group vs hM4D group:Unpaired *t-test* |
| Figure. S1B | Latency of photoactivition at 1-Hz, 5-Hz, 10-Hz, 20-Hz | 1-Hz, 5-Hz, 10-Hz, 20-Hz | ChR2 group: n = 7; mCherry group: n = 4 | 1. Hz: P = 0.6687; 2. Hz: P < 0.0001; 3. Hz: P < 0.0001;   20-Hz: P < 0.0001 | Unpaired *t-test* |
| Figure. S2C | Averaged ΔF/F in REM and Wake | REM/Wake | n = 10 trials from 6 mice | P = 0.0053 | Paired *t-test* |
| Figure. S2E | Peak ΔF/F in spontaneous REM and averaged ΔF/F in REM to Wake drop | Spontaneous in REM; REM to Wake drop | n = 10 trials from 3 mice | P = 0.0049 | Paired *t-test* |
| Figure. S2J | State of REM change in probability | mCherry group; ChR2 group | mCherry group: n = 4 mice; ChR2 group: n = 6 mice | REM: P = 0.9110;  NREM: P = 0.4938;  Wake: P = 0.5019 | Unpaired *t-test* |
| Figure. S3D | Time spent in REM, NREM and Wake state | REM, NREM and Wake | mCherry group: n = 4; hM4D group: n = 8 | Wake state, mCherry-CNO vs hM4D-CNO: P = 0.0388.  Wake state, hM4D-saline vs hM4D-CNO: P = 0.0026.  NREM state, mCherry-CNO vs hM4D-CNO: P = 0.0298.  NREM state, hM4D-saline vs hM4D-CNO: P = 0.0026 | Wake state, mCherry-CNO vs hM4D-CNO: Unpaired *t-test*.  Wake state, hM4D-saline vs hM4D-CNO:Paired *t-test*.  NREM state, mCherry-CNO vs hM4D-CNO: Unpaired *t-test*.  NREM state, hM4D-saline vs hM4D-CNO: Paired *t-test* |
| Figure. S4C | Firing rates | 40dB SPL; 50dB SPL; 60dB SPL; 70dB SPL; 80dB SPL | n = 11 cells | R^2^ = 0.5918  P < 0.0001 | Linear regression |
| Figure. S4F | Firing rates with frequency normalized | 40dB SPL; 50dB SPL; 60dB SPL; 70dB SPL; 80dB SPL | n = 10 cells | R^2^ = 0.6558  P < 0.0001 | Linear regression |
| Figure. S4G | Firing rates with intensity normalized | 40dB SPL; 50dB SPL; 60dB SPL; 70dB SPL; 80dB SPL | n = 10 cells | R^2^ = 0.0776  P = 0.0501 | Linear regression |
| Figure. S4K | Peak ΔF/F of sound-induced in different sound level in awake mice | 40dB SPL; 50dB SPL; 60dB SPL; 70dB SPL; 80dB SPL | n = 5 mice | R^2^ = 0.4458  P = 0.0003 | Linear regression |
| Figure. S5A | Peak ΔF/F in NREM to Arousal and NREM unchanged trials in 40dB SPL sound stimulus | NREM to Arousal ; NREM unchanged | n = 8 mice | P = 0.0004 | Paired *t-test* |
| Figure. S5B | Peak ΔF/F in NREM to Arousal and NREM unchanged trials in 50dB SPL sound stimulus | NREM to Arousal ; NREM unchanged | n = 8 mice | P < 0.0001 | Paired *t-test* |
| Figure. S5C | Peak ΔF/F in NREM to Arousal and NREM unchanged trials in 60dB SPL sound stimulus | NREM to Arousal ; NREM unchanged | n = 8 mice | P = 0.0002 | Paired *t-test* |
| Figure. S5D | Peak ΔF/F in NREM to Arousal and NREM unchanged trials in 80dB SPL sound stimulus | NREM to Arousal ; NREM unchanged | NREM to Arousal: n = 8 mice  NREM unchanged: n = 5 mice | P = 0.0298 | Unpaired *t-test* |
| Figure. S5E | Peak ΔF/F in REM to Arousal and NREM unchanged trials in 40dB SPL sound stimulus | REM to Arousal ; REM unchanged | REM to Arousal: n = 2 mice  REM unchanged: n = 3 mice | P = 0.4893 | Unpaired *t-test* |
| Figure. S5F | Peak ΔF/F in REM to Arousal and NREM unchanged trials in 50dB SPL sound stimulus | REM to Arousal ; REM unchanged | n = 3 mice | P = 0.9608 | Paired *t-test* |
| Figure. S5G | Peak ΔF/F in REM to Arousal and NREM unchanged trials in 60dB SPL sound stimulus | REM to Arousal ; REM unchanged | n = 3 mice | P = 0.8658 | Paired *t-test* |
| Figure. S5H | Peak ΔF/F in REM to Arousal and NREM unchanged trials in 70dB SPL sound stimulus | REM to Arousal ; REM unchanged | n = 3 mice | P = 0.4508 | Paired *t-test* |
| Figure. S5I | Peak ΔF/F in REM to Arousal and NREM unchanged trials in 80dB SPL sound stimulus | REM to Arousal ; REM unchanged | n = 3 mice | P = 0.2217 | Paired *t-test* |
| Figure. S6C | c-Fos stained cell counts in TeA | Noise exposure group; control group | Noise: n = 5 mice; control: n = 3 mice | P = 0.0035 | Unpaired *t-test* |
| Figure. S6D | c-Fos stained cell counts in BLA/La | Noise exposure group; control group | Noise: n = 5 mice; control: n = 3 mice | P = 0.0015 | Unpaired *t-test* |
| Figure. S6E | c-Fos stained cell counts in LPMR | Noise exposure group; control group | Noise: n = 5 mice; control: n = 3 mice | P = 0.8754 | Unpaired *t-test* |
| Figure. S7D | Peak ΔF/F of TeA-BLA/La circuit in NREM to Arousal and NREM unchanged trials in 70dB SPL sound stimulus | NREM to Arousal; NREM unchanged | n = 7 mice | P = 0.0041 | Unpaired *t-test* |
